# A Multi-Omics Study of Familial Lung Cancer: Microbiome and Host Gene Expression Patterns

**DOI:** 10.3389/fimmu.2022.827953

**Published:** 2022-04-11

**Authors:** Ying Chen, Yunchao Huang, Xiaojie Ding, Zhenlin Yang, Liang He, Mingjie Ning, Zhenghong Yang, Daqian He, Lijuan Yang, Zhangyi Liu, Yan Chen, Guangjian Li

**Affiliations:** ^1^ Department of Thoracic Surgery I, the Third Affiliated Hospital of Kunming Medical University (Yunnan Cancer Hospital, Yunnan Cancer Center), Kunming, China; ^2^ The International Cooperation Key Laboratory of Regional Tumor in High Altitude Area, the Third Affiliated Hospital of Kunming Medical University (Yunnan Cancer Hospital, Yunnan Cancer Center), Kunming, China; ^3^ National Cancer Center/National Clinical Research Center for Cancer, Cancer Hospital, Chinese Academy of Medical Sciences and Peking Union Medical College, Beijing, China; ^4^ Department of Clinical Laboratory, The Third Affiliated Hospital of Kunming Medical University (Yunnan Cancer Hospital, Yunnan Cancer Center), Kunming, China; ^5^ KeRui BioTech Co., Ltd, Kunming, China; ^6^ MeiYin BioTech Co., Ltd, Wuhan, China; ^7^ Cancer Research Institute of Yunnan Province, The Third Affiliated Hospital of Kunming Medical University (Yunnan Cancer Hospital, Yunnan Cancer Center), Kunming, China

**Keywords:** microbiome, familial lung cancer, indoor air pollution, immune, IL17, pollutants-detoxication

## Abstract

**Background:**

Inherited susceptibility and environmental carcinogens are crucial players in lung cancer etiology. The lung microbiome is getting rising attention in carcinogenesis. The present work sought to investigate the microbiome in lung cancer patients affected by familial lung cancer (FLC) and indoor air pollution (IAP); and further, to compare host gene expression patterns with their microbiome for potential links.

**Methods:**

Tissue sample pairs (cancer and adjacent nonmalignant tissue) were used for 16S rRNA (microbiome) and RNA-seq (host gene expression). Subgroup microbiome diversities and their matched gene expression patterns were analyzed. Significantly enriched taxa were screened out, based on different clinicopathologic characteristics.

**Results:**

Our FLC microbiome seemed to be smaller, low-diversity, and inactive to change; we noted microbiome differences in gender, age, blood type, anatomy site, histology type, TNM stage as well as IAP and smoking conditions. We also found smoking and IAP dramatically decreased specific-OTU biodiversity, especially in normal lung tissue. Intriguingly, enriched microbes were in three categories: opportunistic pathogens, probiotics, and pollutant-detoxication microbes; this third category involved Sphingomonas, Sphingopyxis, etc. which help degrade pollutants, but may also cause epithelial damage and chronic inflammation. RNA-seq highlighted IL17, Ras, MAPK, and Notch pathways, which are associated with carcinogenesis and compromised immune system.

**Conclusions:**

The lung microbiome can play vital roles in carcinogenesis. FLC and IAP subjects were affected by fragile lung epithelium, vulnerable host-microbes equilibrium, and dysregulated immune surveillance and response. Our findings provided useful information to study the triple interplay among environmental carcinogens, population genetic background, and diversified lung microbiome.

## Introduction

Lung cancer is the most common cancer diagnosed worldwide and also the world’s leading cause of cancer death (1, 2). Furthermore, lung cancer survival remains poor, owing to diagnosis at a later stage and evolving resistance to standard therapies (3). Theoretically, inherited genetic variants and environmental oncogenic factors are crucial players in lung cancer development; therefore, lung cancer is complex and heterogeneous at the genetic, epigenetic as well as microbiome levels (4–6).

Lung cancer susceptibility could be inherited through generations *via* genetic variants, in the form of familial lung cancer (FLC); and there can be unique characteristics in each subpopulation. Many reported an increased lung cancer risk in FLC populations (7–11); some suggested that FLC has a bigger influence in certain ethnic groups (7, 8); others suggested women have a higher risk than their male relatives (9, 10). In this study, we recruited lung cancer patients from China’s Yunnan Province, Xuanwei/Fuyuan areas, which reported some of the highest lung cancer rates in the world (12–14). Importantly, this subject population has special features: familial lung cancer and indoor air pollution (IAP) caused by coal combustion (12–14). Notably, polycyclic aromatic hydrocarbons (PAHs) released during coal combustion could be potent carcinogens (15, 16).

The lung has the largest surface area in the human body, with gas exchange functions, the lung is inevitably exposed to various environmental microorganisms. The influence of the lung microbiome on lung cancer is still unclear; however, an epidemiological survey indicated an association between repeated antibiotic exposure and increased lung cancer risk (17). The respiratory epithelium act as the first defense line against inhaled environmental ingredients, including chemicals, particles, and microbes, and one study reported that the lung microbiome altered in respiratory diseases like asthma, chronic obstructive pulmonary disease (COPD), and cystic fibrosis (18). On the other hand, many works on colon cancer suggested that certain bacteria were associated with chronic inflammation and subsequently increased risk of colon cancer (19, 20); such as, F. nucleatum can induce infiltration of tumor-promoting myeloid cells to create a pro-inflammatory environment (21), and B. fragilis can secrete endotoxins that cause DNA damage and mutations leading to colon cancer (22).

Herein, we hypothesize that inherited susceptibility, environmental pollutants, and tissue-associated lung microbiome, a “triple interaction”, may influence lung carcinogenesis. The present work was designed to investigate the microbiome in lung cancer patients, affected by familial lung cancer and indoor air pollution in China’s Yunnan Province and find characteristic microbes potentially correlated with the features of each subgroup; furthermore, to compare host lung’s gene expression patterns with their microbiome data to detect potential links between the host “tissue-soil” and the microbial community living therein.

## Materials and Methods

### Patients and Tissue Samples

In this study, we recruited lung cancer patients from China’s Yunnan Province, including Xuanwei/Fuyuan areas, who were affected by familial lung cancer (FLC) and indoor air pollution (IAP) (12–14). Patients were selected from those enrolled in the Department of Thoracic Surgery I of Yunnan Cancer Hospital from Sep. 2016 to Sep. 2019. Subjects were selected based on the following criteria: 1) The high-IAP group was patients from Xuanwei/Fuyuan region of Yunnan Province, who reported using coal for heating or cooking for more than 10 years; 2) The low-IAP group included patients from other areas in the same province, who reported no history of occupational or domestic coal use. In total, 17 high-IAP and 17-low IAP patients were enrolled. 3) Subject with familial lung cancer was defined as individual with three or more first-degree relatives affected by lung cancer, and there were 19 FLC patients. Besides all lung cancer cases, five patients with benign lung tumors were also enrolled as control. All the information was based on self-reports and confirmed by personal medical records.

Clinicopathologic data were documented in the hospital cooperated databank (https://www.linkdoc.com). The TNM stage was reviewed according to the 8th edition of The International Association for the Study of Lung Cancer (IASLC) staging system. Clinicopathologic data were shown in **Tables 1**, **2**, **S1**, **S2**; since some patients were double-positive for FLC and IAP, details for every individual and the sequencing type were in **Table S3**. The majority of patients enrolled had adenocarcinoma (AD) and squamous cell carcinoma (SCC). The study was approved by the Ethical Committees of Yunnan Cancer Hospital (No.KY2019.57). All patients provided informed consent.

**Table 1:** Clinicalpathological characteristics of 34 lung cancer patients divided by FLC.

Variables	Total	Familial lung cancer		*P* value ^a^
		Positive	Negative	
Total number of patients	34	19 (55.9%)	15 (44.1%)	
Gender				0.72
Male	22 (64.7%)	13 (68.4%)	9 (60.0%)	
Female	12 (35.3%)	6 (31.6%)	6 (40.0%)	
Average age: 56 years (range 27 – 70)				0.001
≧50 years	24 (70.6%)	9 (47.4%)	15 (100%)	
<50 years	10 (29.4%)	10 (52.6%)	0 (0.0%)	
Average height: 161 cm (range 97 – 176)				0.72
≧161 cm	22 (64.7%)	13 (68.4%)	9 (60.0%)	
<161 cm	12 (35.3%)	6 (31.6%)	6 (40.0%)	
Average weight: 62 kg (range 42 – 80)				1.0
≧62 kg	19 (55.9%)	11 (57.9%)	8 (53.3%)	
<62 kg	15 (44.1%)	8 (42.1%)	7 (46.7%)	
Blood type				0.44
A	9 (26.5%)	4 (21.1%)	5 (33.3%)	
B	6 (17.6%)	2 (10.5%)	4 (26.7%)	
AB	4 (11.8%)	3 (15.8%)	1 (6.7%)	
O	15 (44.1%)	10 (52.6%)	5 (33.3%)	
Smoking history				0.31
Yes (Current or Ex-smoker)	15 (44.1%)	10 (52.6%)	5 (33.3%)	
Never	19 (55.9%)	9 (47.4%)	10 (66.7%)	
Indoor air pollution (IAP)				0.0004
High	17 (50.0%)	15 (78.9%)	2 (13.3%)	
Low	17 (50.0%)	4 (21.1%)	13 (86.7%)	
Anatomy site				0.30 ^b^
Left lung	15 (44.1%)	10 (52.6%)	5 (33.3%)	
Right lung	18 (52.9%)	8 (42.1%)	10 (66.7%)	
Bilateral lung	1 (3.0%)	1 (5.3%)	0 (0.0%)	
Histology type				0.23 ^c^
Adenocarcinoma (AD)	22 (64.7%)	14 (73.6%)	8 (53.3%)	
Squamous cell carcinoma (SCC)	9 (26.5%)	3 (15.8%)	6 (40.0%)	
Small cell lung cancer	1 (3.0%)	1 (5.3%)	0 (0.0%)	
Others	2 (5.8%)	1 (5.3%)	1 (6.7%)	
Stage				0.83
I	11 (32.4%)	5 (26.4%)	6 (40.0%)	
II	3 (8.8%)	2 (10.5%)	1 (6.7%)	
III	16 (47.0%)	10 (52.6%)	6 (40.0%)	
IV	4 (11.8%)	2 (10.5%)	2 (13.3%)	
Distant organ metastasis				1.0
Present	4 (11.8%)	2 (10.5%)	2 (13.3%)	
Absent	30 (88.2%)	17 (89.5%)	13 (86.7%)	

a For categorical variables, using Fisher’s exact test (2-tailed).

b p value calculated for left/right lung only, other sites are not included.

c p value calculated for AD/SCC only, other types are not included.

**Table 2:** Clinicalpathological characteristics of 34 lung cancer patients divided by IAP.

Variables	Total	Indoor air pollution		*P* value^a^
		High	Low	
Total number of patients	34	17 (50.0%)	17 (50.0%)	
Gender				0.72
Male	22 (64.7%)	12 (70.6%)	10 (58.8%)	
Female	12 (35.3%)	5 (29.4%)	7 (41.2%)	
Average age: 56 years (range 27 - 70)				0.26
≧50 years	24 (70.6%)	10 (58.8%)	14 (82.4%)	
<50 years	10 (29.4%)	7 (41.2%)	3 (17.6%)	
Average height: 161 cm (range 97 - 176)				0.72
≧161 cm	22 (64.7%)	12 (70.6%)	10 (58.8%)	
<161 cm	12 (35.3%)	5 (29.4%)	7 (41.2%)	
Average weight: 62 kg (range 42 - 80)				0.49
≧62 kg	19 (55.9%)	11 (64.7%)	8 (47.1%)	
<62 kg	15 (44.1%)	6 (35.3%)	9 (52.9%)	
Blood type				0.34
A	9 (26.5%)	5 (29.4%)	4 (23.5%)	
B	6 (17.6%)	1 (5.9%)	5 (29.4%)	
AB	4 (11.8%)	2 (11.8%)	2 (11.8%)	
O	15 (44.1%)	9 (52.9%)	6 (35.3%)	
Smoking history				1.0
Yes (Current or Ex-smoker)	15 (44.1%)	8 (47.1%)	7 (41.2%)	
Never	19 (55.9%)	9 (52.9%)	10 (58.8%)	
Familial lung cancer (FLC)				0.00036
Positive	19 (55.9%)	15 (88.2%)	4 (23.5%)	
Negative	15 (44.1%)	2 (11.8%)	13 (76.5%)	
Anatomy site				1.0^b^
Left lung	15 (44.1%)	7 (41.2%)	8 (47.1%)	
Right lung	18 (52.9%)	9 (52.9%)	9 (52.9%)	
Bilateral lung	1 (3.0%)	1 (5.9%)	0 (0.0%)	
Histology type				0.11^c^
Adenocarcinoma (AD)	22 (64.7%)	13 (76.5%)	9 (52.9%)	
Squamous cell carcinoma (SCC)	9 (26.5%)	2 (11.8%)	7 (41.2%)	
Small cell lung cancer	1 (3.0%)	1 (5.9%)	0 (0.0%)	
Others	2 (5.8%)	1 (5.9%)	1 (5.9%)	
Stage				0.17
I	11 (32.4%)	7 (41.2%)	4 (23.5%)	
II	3 (8.8%)	0 (0.0%)	3 (17.6%)	
III	16 (47.0%)	8 (47.1%)	8 (47.1%)	
IV	4 (11.8%)	2 (11.8%)	2 (11.8%)	
Distant organ metastasis				1.0
Present	4 (11.8%)	2 (11.8%)	2 (11.8%)	
Absent	30 (88.2%)	15 (88.2%)	15 (88.2%)	

a For categorical variables, using Fisher’s exact test (2-tailed).

b p value calculated for left/right lung only, other sites are not included.

c p value calculated for AD/SCC only, other types are not included.

Tissue sample pairs including cancer and adjacent nonmalignant tissue of the same patient were immediately separated after resection and independently stored in RNAlater (Sigma, St. Louis, MO, USA), all following sterile operation. (In brief, for the sake of ease “normal” is used to represent “adjacent nonmalignant tissue”, as references appear frequently in the manuscript). A slide was cut from every sample for HE stain. Those containing >60% cancer tissue and <15% necrosis were used for the study. However, not all the tissue parts have enough size for both 16S rRNA sequencing and RNA-seq, and not every sample fits the strict quality control standard for sequencing. Primarily, we chose 24 paired tissue samples, plus unpaired samples: 1 cancer, 6 normal, and 5 benign tumors (adjacent normal) for 16S rRNA. After that, 29 normal tissue samples from the same patient pool were selected for RNA-seq, in order to match the host “tissue-soil” and the microbiome inside. Specific sample information was in **Table S3**.

### 16S rRNA Sequencing for the Microbiome, RNA-Seq for Tissue Gene Expression

The 16S rRNA sequencing for tissue microbiome and RNA-seq for host lung tissue gene expression were performed by BGI-TECH (http://www.genomics.cn). Subsequent data processing and analysis were in **Supplementary Materials**. All sequencing data have been deposited under the BioProject: PRJNA790037 and are publicly available at the location: https://www.ncbi.nlm.nih.gov/sra/PRJNA790037

### Statistical Analysis

The primary comparison using separate cancer/normal data covered FLC, IAP, gender, and smoking groups, significantly different microbes in these groups were screened out respectively. In order to better explore the microbe variation among other characteristics, the data were further analyzed for more details by using combined data, which included FLC, IAP, gender, smoking, age, blood type, anatomy site, histology type, and TNM stage. Because certain parameters had more than two subcategories, and 16SrRNA data were not strictly paired, which might make very small subgroups. To address this problem, we combined normal/cancer tissue data from one patient to generally represent the microbiome of that individual (In total 24 paired subjects; for specific microbes detected only in one tissue type, the taxa and abundance were kept directly; for microbes found in both tissues, the mean value of their abundance were used for that taxa). There were seven subjects who only had cancer or normal tissue sequenced, the one-tissue data was used to represent the patient as an alternative option since one’s normal/cancer tissue shared major microbiome, which could be found in the results. Specific information was in supplementary materials (**Tables S4–S23**).

Briefly, α-diversity indices based on species-level were calculated by Mothur (v1.31.2), suing separated normal/cancer tissue data, which included: observed species, Chao, ace, Shannon and Simpson index. β-diversity analysis based on species level was done by QIIME (v1.80), using separated normal/cancer tissue data; unweighted_unifrac PCoA plots were used to represent the β-diversity results. Significantly different microbes among subgroups were screened out by the Wilcox test (applied for 2 subgroups) and Kruskal test (applied for more than 2 subgroups). Differentially expression genes (DEG) between subgroups were identified by DEGseq algorithms. R studio (R v4.1.1) was mainly used to analyze the data, including Venn, PCA, LEFse, PLSDA, PERMANOVA, Wilcox test, and Kruskal test. Statistical analysis for patient clinicopathologic data was evaluated by Fischer’s exact test using SPSS 22.0 (SPSS Institute, Chicago, IL, USA). P<0.05 (two-sided p-value) was considered to be significant.

## Results

### The Overall Landscape of Lung Cancer Microbiome: The Same Dominant Species With a Variety of Subgroup-Specific Microbes in Low Abundance

**Figure 1:**
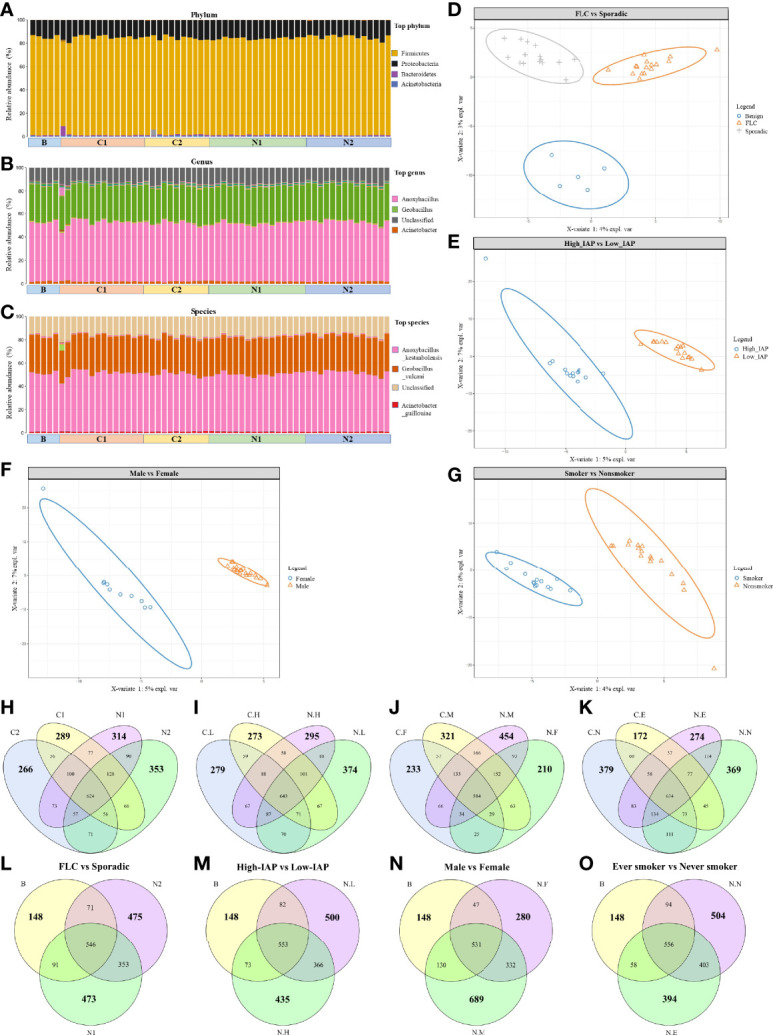
Overall landscape of lung cancer microbiome: the same dominant species and a variety of subgroup-specific microbes in low abundance. Since they were derived from the same organ, all the samples had the same dominant phylum **(A)**, genus **(B)**, and specie **(C)**. Notably, subjects’ microbiome could be separated by PLSDA analysis based on characters: FLC **(D)**, IAP **(E)**, gender **(F)**, and smoking **(G)**. OTU details in four subgroups, subgroup-specific OTU were marked in bigger numbers: **(H)** FLC vs Sporadic lung cancer; **(I)** High-IAP vs Low-IAP; **(J)** Female vs Male; **(K)** smoker vs never smoker. The comparison among subgroup’s normal tissue and the benign control: **(L)** FLC normal vs sporadic normal vs benign; **(M)** High-IAP normal vs Low-IAP normal vs benign; **(N)** Female normal vs Male normal vs benign; **(O)** Ever smokers’ normal vs never smokers’ normal vs benign. C1/N1, cancer/normal tissue of familial lung cancer; C2/N2, cancer/normal tissue of sporadic lung cancer; C.H/N.H, cancer/normal tissue from high indoor air pollution region (High-IAP); C.L/N.L, cancer/normal tissue from low indoor air pollution region (Low-IAP); C.F/N.F, cancer/normal tissue of female lung cancer; C.M/N.M, cancer/normal tissue of male lung cancer; C.E./N.E, cancer/normal tissue from ever smokers; C.N/N.N, cancer/normal tissue from never smokers; B, benign tumor.

In this section, we first depicted the major features of our lung cancer microbiome, the characters of each subgroup would be described specifically in the following sections. In total 60 samples, 2724 OTU were identified (Operational Taxonomic Units); all the tissues had similar OTU, (Average: 319; Range: 251-398; SD 31). Because all samples derived from the same organ—the lung—the dominant microbial communities were the same, and were adapted primarily to the overall lung environment. All subjects had the same dominate phylum, genera and species: in phylum (**Figure 1A**): Firmicutes~83%, Proteobacteria~15%, Bacteroidetes~1%, Actinobacteria~0.34%; in genera (**Figure 1B**): Anoxybacillus~50%, Geobacillus~31%, unclassified~13%, Acinetobacter~2%; and in species (**Figure 1C**): Anoxybacillus _kestanbolensis, Geobacillus _vulcani, Acinetobacter _guillouiae. That was quite different from what was previously found in NCI-MD and TCGA lung cancer, which included totally 1117 tumor/252 normal samples (23). First, our study and their work found the same main phylum but with greatly varied ratio: in NCI-MD/TCGA data, Proteobacteria~60-70% was the dominate phylum, followed by Acinetobacter~13-26% and Firmicutes only 13-20%, Bacteroidetes~3-5%. Second, in NCI-MD/TCGA data, genera Burkholderia, Pseudomonas, Cupriavidus, Streptococcus, Acidovorax were the most abundant in both AD and SCC. Besides, the genera we found were also different from what reported dominate the normal lung: Prevotella, Veillonella, and Streptococcus (24).

Even having the same dominant species, our subjects’ microbiome could be separated by PLSDA analysis based on characteristics: FLC (**Figure 1D**), IAP (**Figure 1E**), gender (**Figure 1F**), and smoking (**Figure 1G**), suggesting these features could influence lung microbiome in certain ways, and there must be some microbes that made each subgroup different. As a result, in the following sections, we combined clues from three indices: subgroup-specific OTU number, α-diversity, and β-diversity to collectively analyze and describe the special characters of each subgroup. Here we fist gave a general description on the three indices.

The subgroup-specific OTU number: OTU number that not shared with other subgroups and unique to this population; it was one means numerically reflecting biodiversity of the subgroup. Notably, every subgroup had their specific OTU (Average: 303; Range: 172-454; SD 69), suggesting a variety of subgroup-specific microbes in low abundance. Interestingly, most cancer groups had reduced specific OTU than their normal counterpart: from the total of eight, six groups all had lower cancer-specific OTU, C1:289 < N1:314, C2:266 < N2:353 (**Figure 1H**), C.L:279 < N.L:374, C.H:273 < N.H:295 (**Figure 1I**), C.M:321 < N.M:454 (**Figure 1J**), and C.E:172 < N.E:274 (**Figure 1K**), while only two groups had slightly higher cancer-specific OTU, C.F:233 > N.F:210 (**Figure 1J**) and C.N:379 > N.N:369 (**Figure 1K**), suggesting generally decreased specific-OTU biodiversity in cancer tissues. Additionally, the benign group, from lungs with no cancer, seemed to show a much lower subgroup-specific OTU than all lung cancer and normal tissues, B:148 < N1:473, B:148 < N2:475 (**Figure 1L**), B:148 < N.L:500, B:148 < N.H:435 (**Figure 1M**), B:148 < N.F:280, B:148 < N.M:689 (**Figure 1N**), B:148 < N.N:504, and B:148 < N.E:394 (**Figure 1O**) This suggests “cancer-free lungs” could possibly have a quite different microbiome than the lungs with cancer; as a result, we did not use the benign group frequently in later comparisons. The subgroup-specific OTU number would also help to explain special features of each subgroup, that would be mentioned in the following sections.

**Figure 2:**
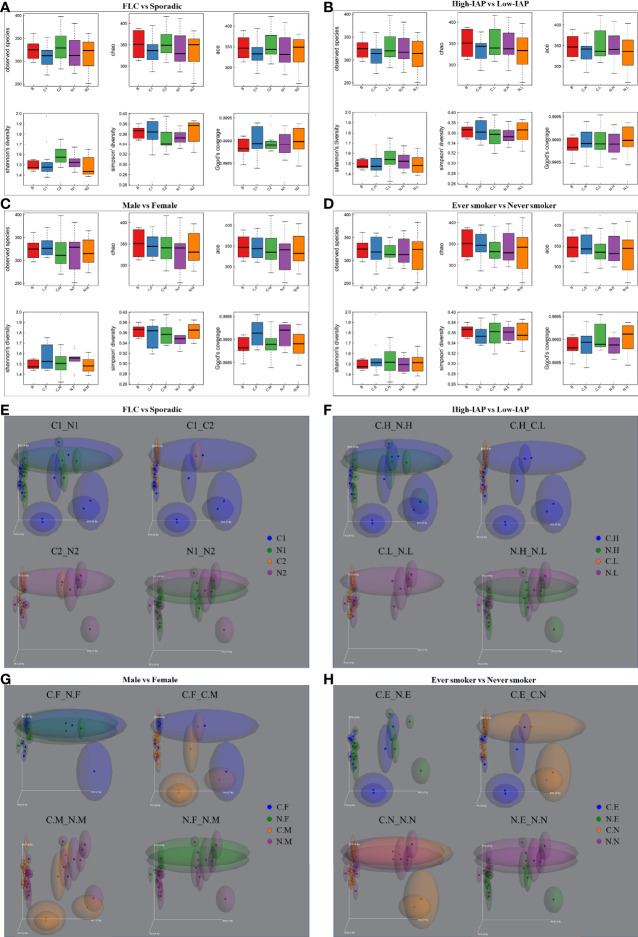
The α-diversity and β-diversity of our subject population. The α-diversity included several indices: observed species, Chao, ace, Shannon, and Simpson. The complexity of the sample is proportional with the first four values, while having a negative correlation with Simpson value. Observed species, Chao, and ACE value can reflect the species richness of the community. Shannon and Simpson values also reflect the species diversity of the community, affected by both specie richness and species evenness, that is the two values also consider the abundance of each species. With the same species richness, the greater the species evenness, the greater the community diversity; that is the bigger the Shannon or the smaller the Simpson value. The Good’s coverage index reflects whether the sequencing results represent the real situation of microbes in the sample. Here, all the groups had a coverage of around 0.999, appropriately reflecting the real situation of the sample’s microbiome. The α-diversity for four groups: **(A)** FLC vs Sporadic lung cancer; **(B)** High-IAP vs Low-IAP; **(C)** Female vs Male; **(D)** Smoker vs never smoker. Five lines from bottom to top is the minimum value, the first quartile, mean, the third quartile, and the maximum value, and the abnormal value is shown as ‘o’. In β-diversity, uniFrac uses the system evolution information to compare the composition of community species between samples. Since different microbes were detected in low abundance, β-diversity was analyzed by unweighted_unifrac, which didn’t calculate the abundance of sequences. The β-diversity for four groups: **(E)** FLC vs Sporadic lung cancer; **(F)** High-IAP vs Low-IAP; **(G)** Female vs Male; **(H)** Smoker vs non-smoker. Since all the samples were lung tissues, all of them had the same dominant phylum, genera, and species, that represented the basic microbiome of the organ lung. As a result, α-diversity and β-diversity based on all species did not reach statistical significance. Although not significant, visible differences could be found among subgroups. These differences are mainly caused by those in low abundance and subgroup-specific microbes, even those ones not dominant; these microbes could still have their influence on the lung microenvironment. C1/N1, cancer/normal tissue of familial lung cancer; C2/N2, cancer/normal tissue of sporadic lung cancer; C.H/N.H, cancer/normal tissue from high indoor air pollution region (High-IAP); C.L/N.L, cancer/normal tissue from low indoor air pollution region (Low-IAP); C.F/N.F, cancer/normal tissue of female lung cancer; C.M/N.M, cancer/normal tissue of male lung cancer; C.E./N.E, cancer/normal tissue from ever smokers; C.N/N.N, cancer/normal tissue from never smokers; B, benign tumor.

The α-diversity and β-diversity evaluate the complexity of the whole microbiome. Since all our samples had the same dominate phylum, genera, and species, so the α-diversity and β-diversity based on all microbes did not reach statistical significance. Although not significant, visible difference could be found among subgroups in their α-diversity and β-diversity. These differences were mainly caused by microbes in low-abundance and subgroup-specific microbes; even though not the dominant ones, these microbes could still have an influence on lung microenvironment. For α-diversity, in **Figure 2A**: subgroup C2 mean value was some higher than C1, N1 in all 5 indices: observed species, Chao, ace, Shannon, Simpson; and C2 was clearly higher than C1, N1, and N2 in Shannon and Simpson value; reflecting that C2 may harbor a more diversified microbiome than C1, N1, and N2. (The sample biodiversity is proportional with: observed species, Chao, ace, and Shannon values, while it has an negative correlation with Simpson value. Observed species, Chao and ace value can reflect the species richness of sample. Shannon and Simpson value reflect the sample diversity affected by both specie richness and species evenness, that is the two also consider the abundance of every species. With the same species richness, greater species evenness and higher biodiversity means a bigger Shannon or smaller Simpson value. The Good’s coverage index reflects whether the sequencing results represent the real situation of microbes in the sample. Here, all our subgroups had coverage around 0.999, and thus appropriately reflected the real situation of the microbiome of the sample. The related α-diversity data were in supplementary materials, **Tables S24–S27**. In following sections, we describe α-diversity difference for **Figures 2B–D**, which reveals the special features of each subgroup gradually.

For β-diversity, since different microbes were in low abundance, in order to reflect the difference induced by less abundant microbes, we used unweighted_unifrac PCoA plots, which looked for how much the two subgroups could overlap with each other, or how much they separated apart. In other words: the more overlap, the more similar the two groups; the less overlap, the bigger their difference, or bigger the change. At the least, it was an alternative means to compare how similar the two microbiomes were, or how much the microbiome changed from normal to cancer. For example, in **Figure 2E**, subgroup N1 and N2 showed much apparent overlap, which meant their microbiome were quite similar to each other, for N1 and N2 were both normal lung tissues, so their microbiome could be similar generally. C1 vs N1 had some clear overlap, but some subjects drifted outside the “overlapped area”, which meant when N1 developed into its cancer form C1, the microbiome changed, but with a smaller variance. Compared with C1 vs N1, C2 vs N2 had less overlap, meaning when N2 developed into cancer form C2, the microbiome changed with a bigger variance. For C1 vs C2, they seemed to have the least overlapped area among the four, so the two groups’ cancer tissues may harbor microbiomes with bigger differences. Later, we would describe β-diversity difference for **Figures 2F–H**, which helped to reveal the special features of subgroups.

In the following sections, we combined clues from the 3 indices: subgroup-specific OTU number, α-diversity, and β-diversity to collectively analyze/describe the special characters of each subgroup. For that, we need to gradually cite pictures from **Figures 1**, **2** to describe the related information step by step.

### FLC Microbiome: Low Total Species, Lower Diversity, Less Tend to Change

Importantly, FLC patients had less normal specific OTU compared with sporadic ones (314 vs 353), but they showed a slightly higher cancer-specific OTU (289 vs 266) (**Figure 1H**). Secondly in **Figures 2A**, FLC indeed had lower α-diversity than the sporadic group in both cancer/normal (mean value: C1<C2, N1<N2, in observed species, Chao, ace). For details, FLC cancer not only had less species richness but also low species evenness, while sporadic cancer microbiome seemed to have relatively higher species richness but also better species evenness (mean value: C1<C2, in Shannon, Simpson); FLC normal also had lower species richness, but it may completely miss some rare species found in sporadic normal, thus seemingly better in species evenness, so the Shannon and Simpson diversity were higher than sporadic normal (mean value: N1>N2, in Shannon, Simpson). In **Figure 2E**, β-diversity revealed: FLC normal microbiome showed clear overlap with sporadic normal, suggesting similarity; but their cancer microbiome separated quite considerably, reflecting more difference. Further, the FLC microbiome changed relatively less between cancer/normal, while sporadic microbiome changed considerably more from normal to cancer (C1 vs N1 had some clear overlap, and some subjects outside the overlapped area, it meant when N1 developed into cancer form C1, the microbiome changed with a smaller variance. Comparatively, C2 vs N2 had less overlap, it meant when N2 developed into cancer form C2, the microbiome changed with a bigger variance.). Notably, 68% of FLC patients were men, so the features of the FLC microbiome were not mainly caused by gender, even women’s microbiome showed similar features in the next paragraph. Taken together, the FLC microbiome seemed to be smaller, low-diversity, and inactive to change.

### Female Microbiome: High Total Species, Low Specific Species, Less Prone to Change; Male Microbiome: Low Total Species, High Specific Species, More Likely to Change

Male/female: our female patients had the minimum normal tissue-specific OTU among all normal subgroups (210 vs 314, 353, 295, 374, 454, 274, 369), while male patients had the highest normal tissue-specific OTU in all subgroups (454, the maximum specific OTU) (**Figures 1H–K**). Women also had much lower specific OTU than men in both normal/cancer tissue, (210 vs 454, 233 vs 321) (**Figure 1J**). Compared with the benign control, women also had much lower normal-specific OTU than men (280 vs 689) (**Figure 1N**). Taken together, in normal lung tissue, women tended to have relatively lower specific species, while men have higher specific species. However in **Figure 2C**, our women showed generally higher α-diversity than men in both cancer/normal (mean value: C.F>C.M, N.F>N.M, in observed species, Chao, ace, Shannon); additionally, the clearly higher observed-species mean value in females indicating: women’s microbiome might have more total species than that of men, while male microbiome harbored relatively fewer total species. Accordingly, our men seemed to have a more “concentrated” microbiome than the women (**Figure 1F**). In **Figure 2G** for β-diversity: our female normal microbiome also showed major overlap with male normal, reflecting general similarity; but the female microbiome did not change too much between normal/cancer (N.F vs C.F almost overlapped together); in contrast, our male microbiome changed clearly more from normal to cancer (N.M vs C.M had relatively less overlapped area, also distributed differently), which led to the separation of male/female cancer microbiome (C.M vs C.F). Summarily, our female microbiome was constituted by high total species, but a lot of these microbes might be shared with others, as a result, the women carried lower specific species; it could possibly explain: carrying a wide range of “shared lung microbes”, made female microbiome less prone to change. Notably, 50% of female patients were sporadic, even some features of the female microbiome were similar to FLC, it could not be completely attributed to FLC ratio.

Smoker/nonsmoker, in **Figure 2D**: in normal lung, nonsmokers’ microbiome showed overall higher α-diversity than smokers’ (mean value: N.N>N.E, in all 5 indices), suggesting generally better lung microenvironment; but smokers had slightly higher cancer α-diversity than nonsmokers (mean value: C.E>C.N, in observed species, Chao, ace, Simpson). That related to what was found in β-diversity (**Figure 2H**): Even their normal microbiome had visible overlap with each other; smoker microbiome changed more from normal to cancer (N.E vs C.E had much less overlapped area, also distributed differently), which led to wider separation in smoker/nonsmoker cancer microbiome (C.N vs C.E had much less overlapped area, and distributed quite differently); while nonsmokers’ microbiome seemed to change much less from normal to cancer (N.N vs C.N largely overlapped together). Summarily: if normal lung had more total species and higher α-diversity, reflecting a microbiome with better buffering potential, thus the microbiomes were less likely to change from normal to cancer. Comparatively, less total species and lower normal lung α-diversity might lead to the microbiome being less stable and more likely to change.

Another finding: from normal to cancer, a slightly increased “cancer α-diversity” could be observed in four subgroups: sporadic (**Figure 2A**, mean value C2>N2 in observed species, Shannon, Simpson), low-IAP (**Figure 2B**, mean value C.L>N.L in observed species, Chao, Shannon, Simpson), men (**Figure 2C**, mean value C.M>N.M in Chao, Shannon, Simpson) and smokers (**Figure 2D**, mean value C.E>N.E in all five indices). Accordingly, these four subgroups also showed relatively larger β-diversity change from normal to cancer (**Figure 2E**, C2 vs N2. **Figure 2F**, C.L vs N.L. **Figure 2G**, C.M vs N.M. **Figure 2H**, C.E vs N.E. all showed relatively less overlapped area or different distribution, compared with their counterpart). One assumption was: some cancer microbiome tended to have more species richness and better species evenness; since host “cancer soil” may support certain microbes’ colonization and reproduction; resulting in higher cancer α-diversity. Potentially, the slight increase in cancer-associated species may reflect the elevated chaos in the cancer tissue itself, which carried various somatic mutations. Other groups also reported increased microbiome richness and diversity in cancer tissue (23, 25).

### Smoking and IAP Decreased Specific-OTU Biodiversity, Especially in Normal Lung

The influence of smoking and IAP on biodiversity appeared complex. Compared with low-IAP patients, the High-IAP group seemed to have slightly lower cancer α-diversity, but higher normal α-diversity (**Figure 2B**, mean value C.H<C.L in observed species, Shannon, Simpson; mean value N.H>N.L in all five indices). Similarly, smokers had a little higher cancer α-diversity, but lower normal α-diversity, when compared with nonsmokers (**Figure 2D**, mean value C.E>C.N in observed species, Chao, ace, Simpson; mean value N.E<N.N in all 5 indies). In **Figure 2F** for β-diversity: high-IAP microbiome changed relatively less between cancer/normal (C.H vs N.H largely overlapped together), and high-IAP normal microbiome overlapped greatly with low-IAP normal, but their cancer microbiomes were clearly different (C.H vs C.L quite less overlapped area); since low-IAP microbiome changed apparently more from normal to cancer (N.L vs C.L very small overlap area). However, considering subgroup-specific OTU made that problem much clearer: smoking and IAP dramatically decreased specific-OTU biodiversity, especially in normal lung tissues. In the normal part, high-IAP specific OTU had a big decrease compared to the low-IAP group (**Figure 1I**: 295 vs 374); and smokers specific OTU dropped much more than nonsmokers (**Figure 1K**: 274 vs 369). Similarly in the cancer part, IAP only made a slight specific OTU change (**Figure 1I**: 273 vs 279), but smoking caused the biggest specific OTU drop (**Figure 1K**: 172 vs 379). Additionally, compared with the benign control, the low-IAP group and nonsmokers both showed higher normal specific OTU than their counterparts (**Figure 1M**: 500 vs 435, **Figure 1O**: 504 vs 394). Conclusively, pollution inside the lung microenvironment was noxious to the “host lung ecosystem”. Generally, pollutants from smoking and coal burning could influence the microbiome of oral, lung, and gut, leading to various diseases (26, 27), *via* directly changing microenvironmental oxygen, pH, and chemical composition; affecting the immune system (28), or promoting colonization of specific taxa through biofilm formation (26, 29).

### Microbes Enriched or Dropped in Lung Cancer Tissue

Among all subgroups, some genera increased universally or frequently in cancer tissue; including Staphylococcus, Capnocytophaga, Lachnoanaerobaculum, Fusobacterium, Oligella, Rubellimicrobium, Marinococcus Sphingomonas, and Sphingopyxis. Firstly, Staphylococcus is commonly found in the environment, and is normal human flora (30); Capnocytophaga, Lachnoanaerobaculum, and Fusobacterium are normal mouth flora; species from these four genera are known to cause opportunistic infection, especially in immune-compromised hosts (30–33). Moreover, Fusobacterium nucleatum is reported to be prevalent in colorectal cancer (33). Oligella ureolytica was identified as a sporadic colonizer in the respiratory tract of cystic fibrosis patients (34), and also reported in bloodstream infection of lung cancer patients (35). In contrast, Rubellimicrobium are mainly isolated from soil and are also found in the air (36); Marinococcus usually lives in high-salinity environments like the sea, salt lakes, and salt mines (37); its accumulation in cancer tissue might be due to salt body fluid effusion. Intriguingly, Sphingomonas and Sphingopyxis are soil microbes; both are famous for their degradation of PAHs, pesticide, and organometallic compounds (38–41). Therefore, enrichment of these microbes in subjects exposed to IAP indicated: PAHs and other pollutants accumulated in patients’ lungs provided a substrate for the microbes, and possibly support their growth.

On the other hand, some genera decreased or depleted in cancer tissue, presenting a different trend: we found Comamonas often decreased in cancer; while species of Comamonas could cause opportunistic infection but are also known to degrade PAHs and are tolerant to heavy metals (42, 43). Additionally, Peptococcus were not detected in cancer tissue but only found in normal tissue; but previous work suggested Peptococcus is also an opportunistic pathogen (44). Taken together, since both were not likely to be probiotics, their abundance drop in cancer tissue might be explained as being out-competed by other microbes. Finally, some genera were only enriched in certain groups, different genera and species were listed in **Tables S28–S39**.

### Microbes Showed Special Features in Familial Lung Cancer

There were microbes highlighted in the FLC group (**Table S28**). From normal tissue to cancer, Capnocytophaga, Fusobacterium rose dramatically in FLC cancer (about 5X and 42X); meanwhile, Sphingomonas, Sphingopyxis both increased apparently in FLC cancer (about 1.3X and 1.4X); reflecting that they were more likely to accumulate in FLC cancer tissue. Additionally, Gemmata was only found in the FLC population from both cancer/normal; it mainly lives in soil, but has also been detected on human skin, so it could be an opportunistic pathogen (45). Rhodococcus decreased in FLC but increased in the sporadic group; some could cause opportunistic infection (46), while its species are also used to degrade environmental pollutants (47).

For further investigation, permutational multivariate analysis of variance (PERMANOVA) was used to process the combined normal/cancer tissue data, and the significantly different microbes were listed in **Table S29**. Interestingly, the genera Staphylococcus, Rubellimicrobium, Oligella, Comamonas, and Sphingomonas were the same as detected in separate cancer/normal data; while Alteromonadales and Acetobacteraceae were newly found. Only staphylococcus was less abundant in the FLC group, the others were all elevated in the FLC population, especially PAHs-degrading Comamonas and Sphingomonas.

### Microbes Elevated in Patients Exposed to Indoor Air Pollution

Like the FLC population, from normal to cancer, Fusobacterium increased quite dramatically in high-IAP cancer than low-IAP (about 24X vs 4X). Notably, some genera only enriched in IAP-related groups (**Table S30**). Firstly, Selenomonas, Thermomonas rose in cancer for both IAP groups, and the two genera were much more abundant in high-IAP normal lung. Selenomonas was reported in the oral microbiome of systemic diseases (48); Thermomonas mainly isolated from soil/sediment, which may have pollutants-degradation potential (49, 50); suggesting they were likely to be opportunistic pathogen flourished in high IAP environment. Secondly, we found Acidovorax, Butyricicoccus decreased in cancer for both IAP groups; while others reported Acidovorax were enriched in smokers’ SCC (23); Butyricicoccus mainly in the gut microbiome, and were reported in colorectal cancer (51). Thirdly, Actinomyces (often in human infection (52)) and Rhodococcus dropped in high-IAP cancer, but rose in Low-IAP cancer; these changes reflected the dynamic complexity of the lung cancer microbiome.

### Microbes Different Between Men/Women and Smokers/Nonsmokers

Notably, Comamonas were 1.4~2 times higher in nonsmokers in both tissue types; from normal to cancer, Sphingomonas dropped in both men and smokers’ cancer but increased in women and nonsmokers’ cancer, so its abundance was 2~3 times higher in women and nonsmokers’ cancer, compared to their counterparts respectively (**Tables S31, 32**). Taken together, these PAHs-degrading microbes tended to accumulate in female or nonsmoker groups. In addition, Capnocytophaga was nearly 9 times more abundant in female normal tissue than males’. Vagococcus was only found in the gender group, it rose in cancer of both genders, and was higher in female cancer. Actually, this genus is often found in animals and is rarely reported in human infection (53).

### Microbiome Varied in Age, Blood Type, Anatomy Site, Histology Type, and TNM Stage

**Figure 3:**
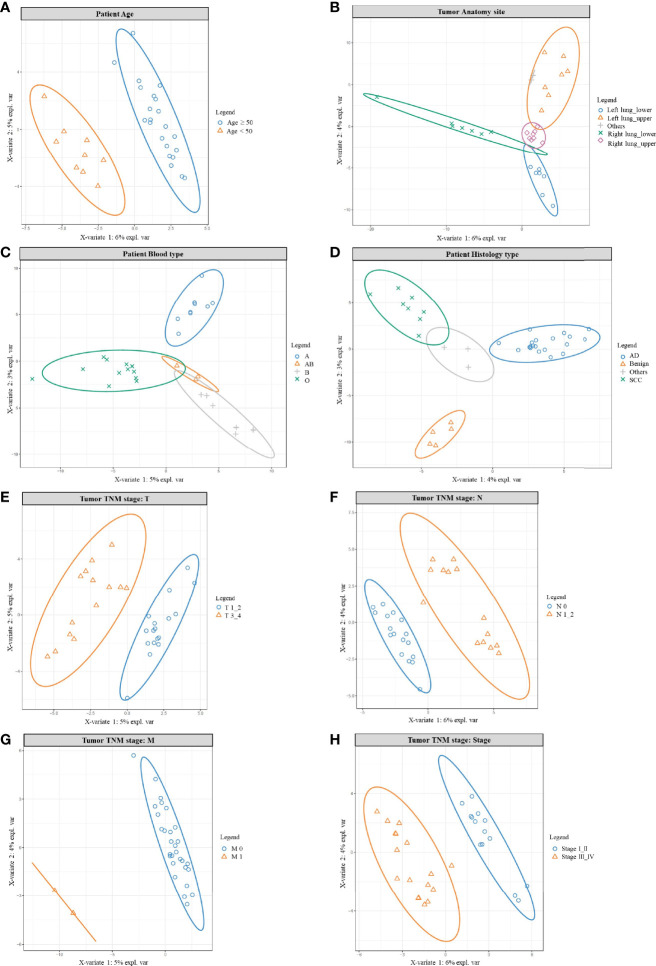
PLSDA results for age, anatomy site, blood type, histology, and TNM stage. PLSDA analysis for patients’ age **(A)**, anatomy site **(B)**, blood type **(C)**, histology type **(D)**, and TNM stage: T stage **(E)**, N stage **(F)**, M stage **(G)**, Tumor stage **(H)**. The combined normal/cancer tissue data from one patient were used to generally represent the microbiome of the individual.

In order to better understand the microbe variation among other characteristics the data was further analyzed, which covered: age, blood type, anatomy site, histology type, and TNM stage (**Figure 3**). The combined normal/cancer tissue data from one patient were used to generally represent the microbiome of the individual, and the significantly different microbes were listed in **Tables S33-S39**. These microbes were mainly found in the environment, and many of them were opportunistic pathogens.

Initially, we found wider genera were more likely to accumulate in patients older than 50 years, while younger patients (<50 years) shared a smaller variety (**Table S33** and **Figure 3A**). For example, opportunistic pathogen Actinomyces, Corynebacterium, Deinococcus, Fusobacterium, Mycoplasma were all clearly higher in the older group, in which Deinococcus species were known for their resistance to radiation and oxidative stress (54). On the other hand, Acetobacter, Acinetobacter, Clostridium, Lactococcus, Oscillospira were more abundant in the younger group, in which Acetobacter and Clostridium function in wastewater treatment, helping degrade pollutants (55); gut microbe Oscillospira was described as a future probiotic (56). Other studies also supported age as a parameter helping to shape the lung microbiome (57).

Interestingly, patients’ microbiome could also be grouped by their tumor anatomy site (**Figure 3B** and **Table S34**); it meant: the left/right lung, the upper/lower lobe were like different “terrain”; that was different habitat for their own microbe population. For example, most different genera were more abundant in the right lung, with many higher in the lower lobes. That could be explained by the structure of the organ: the right lung has wider and shorter bronchus, making inhaled particles/aerosol easier to enter the right side; and the lower lobes were more likely to accumulate bigger particles carrying microbes. Actually, considerable regional variation can be found in one lung, because numerous factors would impact bacterial growth: oxygen tension, pH, temperature, blood perfusion, alveolar ventilation, epithelial cell structure, deposition of inhaled particles, plus concentration and behavior of immune cells (58–60). Besides, another PAHs-degrading Novosphingobium (61) was also detected but seemed to prefer the upper lobes. In addition, we noted patients’ microbiome varied among different blood types (**Figure 3C**, **Table S35**), which consisted with others work: blood type could contribute to shaping human microbiome, for certain bacteria could use host glycans as receptors to adhere to the host, and some liberate host blood glycans as a nutrient (62). Histology type is one major character of cancer, and also inhabited with different microbes (**Figure 3D**, **Table S36**); we found 5 different genera and all were higher in adenocarcinoma. Consistently, other studies also found different microbiomes between lung AD and SCC (23).

Not surprisingly, patients’ microbiome also differed in the TNM stage (**Figures 3E–H**, **Tables S37–S39**). Firstly, most opportunistic pathogens rose in the T3_4 group (**Figure 3E**, **Table S37**), only Massilia and probiotic Lactobacillus were more abundant in the T1_2 group. Secondly, similar was seen in the N group; from N0 to N1-2 (**Figure 3F**, **Table S38**), increased genera mainly cause opportunistic infection. Only Blastomonas, Bradyrhizobium, Kaistobacter were higher in the N0 group; among them, Bradyrhizobium, and Kaistobacter were reported to play important roles in pollutant degradation/detoxication, including PAHs and heavy metals (63, 64). Even there were only two M1 patients, their microbes’ composition still drifted outside the M0 population (**Figure 3G**). Finally, opportunistic pathogens increased in stage III_IV (**Figure 3H**, **Table S39**); but Blautia, Cloacibacterium, and probiotic Lactobacillus were higher in stage I_II patients. In which gut microbe Blautia was reported as potential probiotics (65), and Cloacibacterium were able to detoxify heavy metals by producing extracellular polymers (66). In conclusion, for the earlier stage, we found relatively higher probiotics and pollutants-detoxication genera; while in the later stage opportunistic pathogens rose in abundance and variety. Similarly, other studies also noted differential abundance between stage I–IIIA and IIIB–IV, with later-stage having more enriched oral commensals, such as Haemophilus, Fusobacterium, Gemella, etc. (67).

### Host Normal Lung Gene Expression Patterns: No Significant Overall Expression Difference; With Typical DEGs in Host-Microbes Symbiotic Interactions, Chemical Carcinogenesis, Dysregulated Immune Reaction

**Figure 4:**
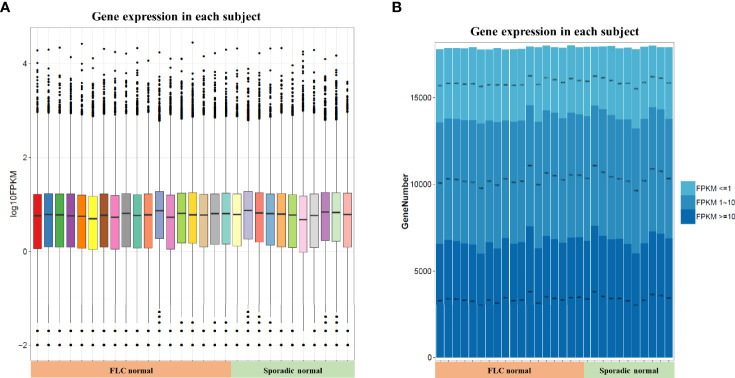
Host normal lung tissue gene overall expression. **(A)** Box-plot of overall gene expression in each subject. X-axis represented each sample, in the related subgroup. Y-axis represented the log_10_FPKM value. Five lines from bottom to top is the minimum value, the first quartile, mean, the third quartile, and the maximum value, and the extreme values were shown as black dots. **(B)** Gene expression distribution in each subject. Which showed the gene amount under three different FPKM ranges (FPKM <= 1、FPKM 1~10、FPKM >= 10)X-axis represented each sample, in the related subgroup. The Y-axis represented the gene amount; the dark color meant the high expression level which FPKM value >= 10, while the light color meant the low expression level which FPKM value <= 1. FPKM: Fragments Per Kilobase per Million, FPKM is a unit used in RNA-seq, which represents gene expression level; the bigger FPKM value, the higher gene expression level.

**Figure 5:**
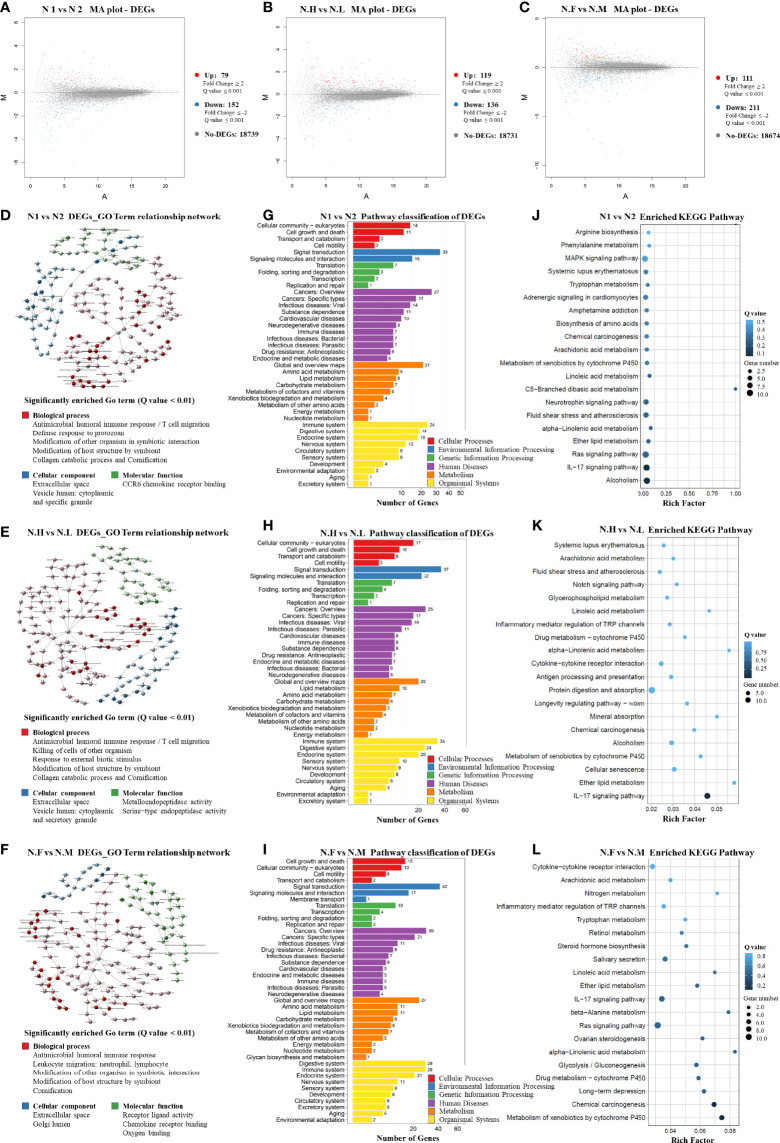
Host normal lung tissue differentially-expressed-gene patterns (1). M-A plot of differentially expressed genes (DEGs) in three groups: **(A)** FLC vs Sporadic lung cancer; **(B)** High-IAP vs Low-IAP; **(C)** Female vs Male (2). DEGs GO Term relationship network in three groups: **(D)** FLC vs Sporadic lung cancer; **(E)** High-IAP vs Low-IAP; **(F)** Female vs Male. The detailed large figures were in supplementary material, **Figures S5–7** (3). Pathway classification of DEGs in three groups: **(G)** FLC vs Sporadic lung cancer; **(H)** High-IAP vs Low-IAP; **(I)** Female vs Male (4). Enriched KEGG Pathway in three groups: **(J)** FLC vs Sporadic lung cancer; **(K)** High-IAP vs Low-IAP; **(L)** Female vs Male. N1, normal tissue of familial lung cancer; N2, normal tissue of sporadic lung cancer; N.H, normal tissue from high indoor air pollution region (High-IAP); N.L, normal tissue from low indoor air pollution region (Low-IAP); N.F, normal tissue of female lung cancer; N.M, normal tissue of male lung cancer.

In order to compare host “tissue-soil” and the microbiome live inside, 29 normal tissue samples from the same patient pool were selected for RNA-seq, the gene expression pattern in each group was illuminated. In our cohort, a total of 19348 genes were detected; since all samples were normal lung tissue, which meant the genes expression need to maintain “generally normal” tissue structure and functions; thus, no significant difference was observed based on the overall gene expression. In **Figure 4A**, across FLC and sporadic patients, every individual had very similar gene-expression-bar and mean-expression levels. In **Figure 4B**, every subject showed similar gene expression level distribution for high level (FPKM ≥ 10), middle (FPKM 1~10), and low level (FPKM ≤ 1), no matter they were FLC or sporadic. As no major difference, we focused on finding genes with smaller variations among subgroups. In gene expression analysis, subgroups FLC, IAP, and gender were included. Because the comparisons were between normal tissues, only a few differentially expressed genes (DEGs) were found in each group (**Figures 5A–C** and **Tables S40–S42**). They were: FLC vs sporadic, up 79 down 152; high-IAP vs low-IAP, up 119 down 136; female vs male, up 111 down 211; with DEGs, Gene Ontology (GO) and KEGG pathway classification and functional enrichment were performed (**Figures 5D–L**).

Host gene expression seemed to have less diversity than their microbiome since individual subjects could have different microbiomes, but all patients shared the same set of human genes. As a result of GO analysis, DEGs were enriched in quite representative biological processes universally in 3 subgroups (**Figures 5D–F**): (1) “Antimicrobial humoral and cell immune response”. This is an important interaction between lung microbiome and host tissue, basically to clean the lung environment, control microbe community, and maintain equilibrium. On the host side, necessary interaction with normal microbiome kept the body immune system healthy and functional (68). However, a biased immune response could be an accomplice: one study suggested commensal microbiota promote lung cancer development *via* γδ T Cells, by tumor-promoting inflammation (69). (2) “Modification of host structure and modification other organisms in symbiotic interaction”. Reflecting microbes and host cells dynamically adjusting their structure, metabolism, activities to adapt to living with each other. This basic process usually is a healthy balance, but once twisted by variables like inhaled carcinogens or hazardous microbes, which cause cell mutations and chronic inflammation, the combined effects could be cancer-promoting. (3) “Collagen catabolic process and cornification”. Collagen is one major component of the extracellular matrix, which provides a surface and nutrients for microbes. However, microbes’ activities like catabolizing extracellular components might damage the epithelial protective barrier, making cells more exposed to environmental carcinogens and infective agents. Cornification is a kind of apoptosis of squamous epithelial cells, and was reported associated with lung cancer prognosis (70). Secondly, the DEGs enriched cellular component included: “extracellular space”, in which most of the symbiotic interaction taken place. “cytoplasmic and specific granule vesicle lumen”, these included secretory and endocytic vesicles; the latter may contain microbes inside, while secretory vesicles from immune cells had a variety of functions in inflammation, recruiting immune cells, killing microbes, etc (68).

In KEGG analysis, DEGs were also enriched in representative pathways similarly in three subgroups. Crucial pathway classification was summarized from **Figures 5G–I**, based on major categories and keywords; which contained: (1) “Cell communication and signal transduction”, which represented the basic cell functions and multi-cell collaborations; actually cell-cell corporations had more advantages in defense against invasive microbes, such as epithelial cells bind tightly to from “defensive barrier”, while immune cells killing/removing harmful microbes. (2) “Cancer”, it has been long known: the adjacent nonmalignant tissue was not strictly normal, the gene expression also betrayed some features of cancer, but the samples still provide useful information. (3) “Infectious disease caused by bacteria, virus, parasites”, that might reflect the infections in cancer patients were complex, cancer could induce an immune-compromised microenvironment (25), led patient increasingly vulnerable to infectious agents. (4) “Xenobiotics biodegradation and metabolism”, indicating cells’ efforts in degrading inhaled carcinogens as well as microbes’ products/toxins, reflecting a very complicated chemical environment the lung cells had to cope with. (5) “Immune and digestive systems” were listed as the top two organismal systems, the immune system played vital roles in controlling microbes, while the digestive system may hold the biggest microbiome in the body, and together might hint at the important equilibrium between the immune system and body microbiome. Specifically, the typical enriched KEGG pathway was summarized from **Figures 5J–L**: (1) “IL-17 signaling pathway”. Actually, IL-17 is overexpressed in a subset of lung cancer patients, it has been considered as an important cytokine in cancer promotion, and was reported to inhibit antitumor immune responses (71); plus, IL17 inflammatory phenotype could be caused by lower airway dysbiosis (67). (2) “Metabolism of xenobiotics by cytochrome P450”. (3) “Chemical carcinogenesis”. These two pathways were related to IAP and smoking conditions, in which subjects were exposed to carcinogens, accompanied by cells’ efforts on metabolic detoxification. (4) “Ras signaling pathway”, which is the famous cancer pathway. Summarily, the KEGG data collectively reflected triple interaction among host, pollutants, and microbiome, host immune response was clearly underlined.

Additionally, there were some enriched biological processes special to certain groups. For example, in the FLC population: “defense response to protozoan” (**Figure 5D**), reflecting FLC patients were more susceptible to opportunistic infections; actually, immune reactions against bacteria, virus, and parasites were all detected. “MAPK signaling pathway” (**Figure 5J**), the famous cancer pathway, was also enriched in the FLC group. “Alcoholism” (**Figure 5J**) might be explained by the drinking habits of some subjects, as a history of drinking was found in the personal medical records of some men. Furthermore, in the IAP subgroup, “Metalloendopeptidase activity” (**Figure 5E**) was detected, which was reported to suppress cancer metastasis (72); and “Notch signaling pathway” (**Figure 5K**), another canonical cancer pathway was enriched. Taken together, most DEGs were enriched in the process covering cancer promotion by chemical carcinogens and dysregulated immune reaction, in which microbes joined at least partly through symbiotic interaction with host epithelial cells and immune system.

## Discussion

The lung microbiome is complex and highly diversified. Ours and others’ findings all suggested that each population may have its own characteristic dominant microbes, depending on the subjects’ source (23, 25, 67, 69). Importantly, a variety of subgroup-specific microbes existed in low abundance and reflected unique features of different subgroups. In a large population, the characters shared by the majority would stand out, but signatures of certain subgroups might be submerged. Our cohort had unique features of familial lung cancer (FLC) and indoor air pollution (IAP), which is a good model to study the complex interaction among fundamental variables in lung cancer etiology: patients’ genetic background, environmental carcinogens, as well as the specific microbiome. Firstly, our FLC microbiome seemed to be smaller, low-diversity, and inactive to change; that matched the features of an unhealthy “ecosystem” (68). In addition, FLC subjects also had features of younger age, later stage, higher tumor malignancy, etc. (73, 74). For both normal and cancer tissue, gene mutations might allow pathogenic species to dominate the community or increase the virulence of other normally commensal microbes; as one study noted: Apc mutation enhances mucosal bacterial adherence (75). Together, the FLC population may suffer from fragile lung epithelium, compromised immune surveillance, and weakened immune response (8, 11, 74). Therefore, we supposed that FLC susceptible factors cause increased lung cancer risks may partly by affecting lung biodiversity, especially in normal lung tissue. The susceptible mutations made the epithelial extracellular matrix “suitable soil” for some genera, while FLC immune systems were unable to clean inhaled microbes effectively; together creating a vulnerable host-microbes equilibrium.

Not surprisingly, we also noted microbiome differences in gender, age, blood type, anatomy site, histology type, TNM stage as well as IAP and smoking conditions; possibly suggesting the microbe community would make adaptive changes to almost any variable. Other studies also supported microbiome could vary among age, blood type, anatomy site, histology, TNM stage, and smoking (23, 26, 27, 57, 62, 67). Naturally, living microbes are sensitive organisms actively respond to their environment, adjusting themselves and making adaptive changes. The lung extracellular matrix and mucus layer are their “habitat”, like soil in the ecosystem, and changes in host “tissue soil” would to a greater or lesser extent influence microbes’ suitable niche or available resources, thus affecting their abundance or clone location. On the other side, chemicals from consistently inhaled air are also a certain “resource” or “toxin” to microbes, inhibiting some genera while benefiting some others. That is why smoking or air pollution could directly affect the lung microbiome. On a large scale, this highly diversified adaptability of tumor microbiome was supported by a study covering 1526 tumors across 7 cancer types, including breast, lung, ovary, pancreas, melanoma, bone, and brain tumors. They found each tumor type had a distinct microbiome composition, and also noted correlations between intratumor bacteria with tumor types, subtypes, smoking status, response to immunotherapy, etc. (25).

Environmental carcinogens are a vital culprit in lung cancer, they could induce genomic DNA mutations in host epithelial cells, making them potentially cancerous. Here, we found smoking and IAP dramatically decreased specific-OTU biodiversity, especially in normal lung tissue, suggesting the hazardous effects also struck the host microbiome. Generally, chemicals and particles from smoking or coal-burning could influence the microbiome of mouth, lung, and gut, leading to various diseases (26, 27); *via* directly changing microenvironmental oxygen, pH, and chemical composition; damaging epithelial cells (76); affecting the immune system (28); or promoting colonization and proliferation of certain taxa through biofilm formation (26, 29). Specifically, these pollutants affect the immune system on many levels: they influence the number and activity of macrophages, neutrophils, eosinophils, NK cells, mast cells, and airway dendritic cells, possibly leading to higher susceptibility to infections (28, 77). Biofilm is microbes’ self-organized polymer structure that insulates themselves from host defense and antibiotics, promoting bacterial persistence (29). One study in colon cancer indicated: biofilm could invade the host epithelial cell layer, increase DNA damage and foster chronic inflammation (75). It is possible that the metabolic advantages of biofilm and increased adherence to the epithelium support certain taxa expansion in a polluted lung environment. Additionally, the anchored biofilm could function as a stronghold for various opportunistic pathogens, which otherwise should be cleaned by the host immune system or antibiotics; as a result, expanding the microbes’ community in the lung, with increasing microbe activities, further damaging lung epithelium and exacerbating host immune burden, like a vicious cycle. Therefore, FLC factors could cause decreased normal lung biodiversity; then, smoking or IAP made even greater dysbiosis in normal lung ecosystems; these all contributed to lung cancer carcinogenesis.

Intriguingly, different microbes were enriched in 3 major categories: opportunistic pathogens, probiotics, and pollutants-detoxication microbes. The 1st category like Staphylococcus, Capnocytophaga, Fusobacterium, etc. often known to cause opportunistic infections. The 2nd included: Lactobacillus, Blautia, Oscillospira, which were recognized or predicted future probiotics, supposed to play a positive role in the microbiome (56, 65). Notably, the third involved: Sphingomonas, Sphingopyxis, Novosphingobium, Comamonas, Rhodococcus, Thermomonas, Bradyrhizobium, Kaistobacter, and Cloacibacterium, which are well-known for their degradation and detoxication of PAHs, pesticide, heavy metals and organometallic compounds (38–43, 47, 49, 50, 61, 63, 64, 66). Importantly, these microbes were frequently enriched in subjects of the FLC and IAP groups. Pollutants or cigarette smoke have been shown to reduce epithelial integrity and cell-cell contact, which can increase susceptibility to respiratory pathogens (76, 78). Further, continual inhalation of PAHs and other pollutants into patients’ lungs possibly provided a substrate for the microbes, and supported their growth. FLC genetic background might also facilitate these genera to colonize, taken together, allowing them to flourish in certain lung cancer microenvironments. Moreover, for younger age and earlier stages, we found relatively higher probiotics and pollutants-detoxication genera. However, in older age and later stage, the former two categories decreased, while opportunistic pathogens rose in abundance and variety. It seemed that pollutants-detoxication microbes were partly helping to save the “polluted lung”. Actually, we considered their existence as a “double-edged sword”: on the one side, they helped to degrade inhaled pollutants, reducing toxin accumulation and alleviating noxious effects on host cells. On the other side, they were not the human normal flora and also not in the “right biological niche”. Additionally, microbes could use host cells’ proteins and carbohydrates as an energy source. So, their nutrient-collecting activities, bacterial components, and pollutants metabolites might still cause stimulation or other hazardous effects on host cells. Moreover, they may be recognized as opportunistic pathogens by the immune system and induce chronic inflammation, which is a cancer-promoting condition (67, 69).

Our RNA-seq supported the conclusions from microbiome analysis, by the “tissue soil” perspective. Most DEGs were enriched in a representative process: “cancer promotion by chemical carcinogens and dysregulated immune reaction”. Firstly, environmental carcinogens could induce mutations in host cells; unfortunately, many cancer patients suffered dysregulated immune systems, that unable to eliminate or contain cancer cells effectively. Likely in our RNA-seq, the active IL17 pathway was frequently highlighted. Multiple studies also supported: increased IL17 production was associated with accelerated lung tumor growth, decreased responsiveness to checkpoint inhibition, and decreased survival (67, 69, 71). Our RNA-seq also indicated: “local microbes took part in carcinogenesis process by symbiotic interaction with host epithelial cell and immune system”. Certain genera might exhibit an adaptive advantage on mutated epithelial cells; as one work reported: some taxa like Acidovorax had a higher abundance in lung cancer with TP53 mutations (23). A group put evidence that: local microbiota provoked inflammation associated with lung cancer by activating lung-resident γδ T cells (69). Others noted: local lung microbiota could trigger host transcriptomic signatures associated with carcinogenesis, like upregulation of IL17, PI3K, MAPK, and ERK pathways; and lower airway dysbiosis led to increased local inflammation (67). Preclinical models also showed that lower airway mucosal inflammation is primarily associated with the composition of the lower airway microbiota rather than the gut or upper airway microbiota (79). The nutrient supply of the airways is abruptly increased during inflammation: increased vascular permeability and mucus production, which resulted in local pockets of anoxia (80) and rising temperature (81), combined with inflammatory by-products, all these could selectively support the colonization & proliferation of certain genera. At the same time, the immune cells kill and clear bacteria with highly varied efficiency (82), creating selection pressure across the bacterial community. Taken together, these dynamic symbiotic interactions would twist the balance toward “cancer-inhibiting” or “cancer-promoting”. In our cohort, RNA-seq highlighted pathways associated with carcinogenesis, like IL17, Ras, MAPK, and Notch, especially in FLC and IAP groups.

Conclusively, the lung microbiome can play vital roles in lung cancer pathogenesis, its symbiotic interaction with an FLC host, and diverse responses to IAP highlighted the complexity of the lung “micro-ecosystem”. Our findings provided useful information to study the intricate interaction between environmental carcinogens, population genetic background as well as diversified lung microbiome.

## Data Availability Statement

The data presented in the study are deposited in the NCBI repository, accession number: PRJNA790037. Link: https://www.ncbi.nlm.nih.gov/sra/PRJNA790037.

## Ethics Statement

The studies involving human participants were reviewed and approved by The Ethical Committees of Yunnan Cancer Hospital (No.KY2019.57). The patients/participants provided their written informed consent to participate in this study.

## Author Contributions

Conception and design: YiC and XD. Foundation support: YH, YaC, and GL. Sample and clinical information collection, sample processing: YiC, LH, MN, ZHY, and DH. Data processing, data analysis and interpretation: YiC, XD, ZLY, LY, and ZL. Writing and revising the article: all authors. All authors contributed to the article and approved the submitted version.

## Funding

This work was supported by the National Science Foundation of China (No: 82060426, 81702274, 81560380, 81960500); Yunnan Health Training Project of High-Level Talent (H-2018025). Doctor Research Foundation of Yunnan Cancer Hospital (No: BSKY201705).

## Conflict of Interest

Author LY is the CEO of KeRui BioTech Co.,Ltd; Author ZYL is the CEO of MeiYin BioTech Co.,Ltd.

The remaining authors declare that the research was conducted in the absence of any commercial or financial relationships that could be construed as a potential conflict of interest.

## Publisher’s Note

All claims expressed in this article are solely those of the authors and do not necessarily represent those of their affiliated organizations, or those of the publisher, the editors and the reviewers. Any product that may be evaluated in this article, or claim that may be made by its manufacturer, is not guaranteed or endorsed by the publisher.

## Supplementary Material

Supplementary Tables 1–3 for clinicopathological characteristics of lung cancer patients;

Supplementary Tables 28–39 for significantly different microbes between groups;

Supplementary Tables 40–42 for significantly different top genes between groups; Supplementary Table for matching patient information to BioProject: PRJNA790037.

## Abbreviations

FLC, familial lung cancer; IAP, indoor air pollution; AD, Adenocarcinoma; SCC, squamous cell carcinoma; SCLC, small cell lung cancer; OTU, Operational Taxonomic Units; DEG, differentially expressed genes.
