# Supplementary material for: A Multi-Omics Study of Familial Lung Cancer: Microbiome and Host Gene Expression Patterns
Source: Front Immunol. 2022 Apr 11;13:827953. doi: 10.3389/fimmu.2022.827953 (PMC9037597; doi:10.3389/fimmu.2022.827953)
Supplement: Supplementary file 1 [file DataSheet_1.docx]

**Supplementary material for Materials and Methods, and Results**

**Subject population background**

Our subject population were recruited from China’s Yunnan Province, certain region here reported some of the highest lung cancer rates in the world, such as Xuanwei / Fuyuan [ref.1-4], and these areas have long been focus of lung cancer studies, including epidemiology, molecular and clinical research. Interestingly, the subject population has two characters: familial lung cancer (FLC) and indoor air pollution (IAP) caused by coal combustion, since local residents used coal for cooking and heating for generations [ref.1-4].

Our previous studies [ref.5,6] on this population already found some of the important clinicopathologic features. Including: younger age, later stage; higher rate of female, adenocarcinoma and other cancer history; as well as gender-specific and age-specific characters. In addition, FLC patients were more likely to develop benign lesions (polyps, nodules, cysts) early in life, especially early-growth of multiple pulmonary nodules; all pointed to increased malignancy in these cancer patients.

Present work is one of the series researches on this representative population, which is a good model to study the complex interactions among fundamental variables in lung cancer etiology: patients’ genetic background, environmental carcinogens as well as specific microbiome. Based on the findings, our ultimate purpose is to find possible means helping lung cancer prevention, susceptibility screen, early-diagnosis and treatment. Such as: finding potential biomarkers for lung cancer risk screen or early-diagnosis, especially for FLC population; using probiotics or their products in preventive strategies; using herb medicine plus probiotics for immune regulation or for hazardous microbe inhibition; any means that may help to reduce lung cancer rate, improve patients’ prognosis and life quality.

**Materials and Methods**

In this study, we recruited lung cancer patients from China’s Yunnan Province, including Xuanwei / Fuyuan areas, which were affected by familial lung cancer (FLC) and indoor air pollution (IAP). In total, 17 high-IAP and 17 low-IAP patients were enrolled; among them, there were 19 FLC patients and 15 sporadic. 5 benign lung tumor cases were also enrolled as control (Table.S3).

Because all surgical samples should meet hospital use first, such as pathological examination and patient tumor record kept in paraffin; only the extra tissue could be used for research. Objectively familial lung cancer tissue samples were relatively rare; as a result, not all the tissues had enough size for both 16S rRNA and RNA-seq. In fact, we do send extra samples for both sequencing, about 6 backup sample pairs, but not every sample fit the strict quality control standard for sequencing; that meant: some pairs only had normal tissue qualified, while some only the cancer part qualified. Of course, paired samples were the best, but in order to analyze valuable samples as much as possible; besides the 24 paired tissue samples, we chose unpaired sample: 1 cancer, 6 normal and 5 benign tumors (adjacent normal) for 16S rRNA. After that, 29 normal tissue samples with good quality and quantity were selected from the same patient pool for RNA-seq. In order to match host “tissue-soil” and the microbiome inside.

In the fine detailed comparison, which included age, blood type, anatomy site, histology type and TNM stage. Since certain parameter had more than two subcategories, and 16SrRNA data were not strictly paired, that would make very small subgroups and the subgroup division would be different between cancer/normal, which became hard to process also less convincing. To address this problem, we combined normal/cancer tissue data from one patient to generally represent the microbiome of that individual. In 24 paired subjects; for specific microbes detected only in one tissue type, the taxa and abundance were kept directly; for microbes found in both tissues, the mean value of their abundance were used for that taxa. There were 7 cancer patients only had cancer or normal tissue sequenced, the one-tissue data was used to represent the patient as an alternative choice, since one’s normal/cancer tissue shared major microbiome, which could be found in the results (Fig.1 A,B,C). Later results proved this strategy work: in FLC, PERMANOVA was used to process the combined normal/cancer data, and the significant different genera: *Staphylococcus, Rubellimicrobium, Oligella, Comamonas, Sphingomonas* were exactly the same as detected by kruskal.test based on the separate cancer/normal data. In addition, by using combined data, the enriched microbes were still in 3 major categories: opportunistic pathogens, probiotics, and pollutants-detoxication genera; basically, similar to what found in separate data. For example, in earlier stage, we found relatively higher probiotics and pollutants-detoxication genera; while in later stage, opportunistic pathogens clearly rose in abundance and variety; the combined data still provided meaningful findings.

**16S rRNA sequencing for microbiome**

- Paired-end reads were generated with Illumina HiSeq platform, then the reads with sequencing adapters, N base, poly base, low quality etc. were filtered out with default parameters.
- If the two paired-end reads overlapped, the consensus sequence was generated by FLASH (Fast Length Adjustment of Short reads, v1.2.11)

1) Minimal overlapping length: 15 bp;

2) Mismatching ratio of overlapped region: <= 0.1

- The high quality paired-end reads were combined to tags based on overlaps, 3425078 tags were obtained in total with 57084 tags per sample on average, and the average length is 252 bp.
- The tags were clustered to OUT (Operational Taxonomic Unit) by scripts of software USEARCH (v7.0.1090), with a 97% threshold.
- OTU representative sequences were taxonomically classified using Ribosomal Database Project (RDP) Classifier v.2.2, using 0.6 confidence values as cutoff.

Databases used for species annotation:

16S rDNA for bacterial: Greengene: V201305; RDP: Release11_5,20160930

- OTU number per sample primarily represents the degree of sample diversity. The tag number of each taxonomic rank (Phylum, Class, Order, Family, Genus, Species) or OTU in different samples were summarized to represent the abundance of each taxa.
- α-diversity indices were calculated by Mothur (v1.31.2), including: observed species, chao, ace, shannon and simpson.
- β-diversity analysis was done by QIIME (v1.80), including: Bray-Curtis，weighted UniFrac，unweighted UniFrac.
- R studio (R v4.1.1) was also used to analyze the obtained data, including: venn, PCA, LEFse, PLSDA, PERMANOVA, wilcox.test, kruskal.test,

**RNA-seq for tissue gene expression**

- The sequencing reads were generated with Illumina HiSeq platform, The average mapping ratio with reference genome is 94.03%, the average mapping ratio with gene is 77.04%; A total of 19,348 genes were detected.
- Clean reads were mapped to reference transcripts using Bowtie2 (v2.2.5), then calculated gene expression level for each sample with RSEM.
- Based on the gene expression level, the DEG (Differentially expression genes) between subgroups were identified, by using DEGseq algorithms.
- With DEGs, we performed Gene Ontology (GO) classification and functional enrichment. In 3 ontologies: molecular biological function, cellular component and biological process.
- With DEGs, we performed KEGG pathway classification and functional enrichment.

**Statistical analysis general summary**

The α-diversity indices based on species level were calculated by Mothur (v1.31.2), suing separated normal/cancer tissue data, which included: observed species, chao, ace, shannon and simpson index. β-diversity analysis based on species level was done by QIIME (v1.80), suing separated normal/cancer tissue data; unweighted_unifrac PCoA plots were used to represent the β-diversity results. Significantly different microbes among subgroups were screened out by wilcox.test (applied for 2 subgroups) and kruskal.test (applied for more than 2 subgroups). Differentially expression genes (DEG) between subgroups were identified by DEGseq algorithms. R studio (R v4.1.1) was mainly used to analyze the data, including: venn, PCA, LEFse, PLSDA, PERMANOVA, wilcox.test, kruskal.test. Statistical analysis for patient clinicopathologic data was evaluated by Fischer's exact test using SPSS 22.0 (SPSS Institute, Chicago, IL, USA). P<0.05 (two-sided p-value) was considered to be significant.

**Subgroup detailed** **information and related analysis (****Table. S4-S23)**

Table. S4-S23 provided the detailed information on each analysis done in the present study. Including: the specific subgroups in each analysis, the related tissue type, data type, total number of patients in each subgroup, and patient’ s individual NO. which could be located in Table. S3 (Table. S3 included every individual’s clinicalpathological characteristics)

**Table.S4 Sequencing type for each subject**

| **Seq-type** | **Tissue type** | **Total no.** | **Sample number in Table. S3** |
| --- | --- | --- | --- |
| 16S | Paired | 24 | F1, F2, F3, F4, F5, F6, F8, F9, F11, F13, F14, F16, F17, F18, S3, S4, S5, S6, S8, S9, S10, S11, S12, S14 |
| 16S | Cancer | 1 | S1 |
| 16S | Normal | 6 | F10, F15, S2, S7, S13, S15 |
| 16S | Benign | 5 | B1, B2, B3, B4, B5 |
| RNA-seq | Normal | 29 | F1, F2, F3, F4, F5, F6, F7, F8, F9, F10, F11, F12, F13, F14, F16, F17, F18, F19, S2, S3, S4, S5, S7, S8, S9, S10, S12, S14, S15 |

**Table.S5 Subgroup detailed information for FLC/Sporadic**

| **Group** | **16S Data** | **Total no.** | **Sample number in Table. S3** |
| --- | --- | --- | --- |
| FLC | Cancer | 14 | F1, F2, F3, F4, F5, F6, F8, F9, F11, F13, F14, F16, F17, F18, |
|  | Normal | 16 | F1, F2, F3, F4, F5, F6, F8, F9, F10, F11, F13, F14, F15, F16, F17, F18, |
| Sporadic | Cancer | 11 | S1, S3, S4, S5, S6, S8, S9, S10, S11, S12, S14, |
|  | Normal | 14 | S2, S3, S4, S5, S6, S7, S8, S9, S10, S11, S12, S13, S14, S15 |

In these 4 subgroups, separated cancer/normal 16S data were used, each group had around 10 subjects or more. The α-diversity indices based on species level were calculated by Mothur (v1.31.2), which included: observed species, chao, ace, shannon and simpson index. β-diversity analysis based on species level was done by QIIME (v1.80), and represented by unweighted_unifrac PCoA plots. (Figures for α-diversity and β-diversity were in Fig.2). Significantly different microbes among subgroups were screened out by kruskal.test (applied for more than 2 subgroups). (Table.S28)

**Table.S6 Subgroup detailed information for High-IAP/Low-IAP**

| **Group** | **16S Data** | **Total no.** | **Sample number in Table. S3** |
| --- | --- | --- | --- |
| High-IAP | Cancer | 13 | F1, F2, F3, F4, F5, F8, F9, F11, F14, F16, F17, S5, S6 |
|  | Normal | 14 | F1, F2, F3, F4, F5, F8, F9, F11, F14, F15, F16, F17, S5, S6 |
| Low-IAP | Cancer | 12 | F6, F13, F18, S1, S3, S4, S8, S9, S10, S11, S12, S14 |
|  | Normal | 16 | F6, F10, F13, F18, S2, S3, S4, S7, S8, S9, S10, S11, S12, S13, S14, S15 |

In these 4 subgroups, separated cancer/normal 16S data were used, each group had around 10 subjects or more. The α-diversity indices based on species level were calculated by Mothur (v1.31.2), which included: observed species, chao, ace, shannon and simpson index. β-diversity analysis based on species level was done by QIIME (v1.80), and represented by unweighted_unifrac PCoA plots. (Figures for α-diversity and β-diversity were in Fig.2). Significantly different microbes among subgroups were screened out by kruskal.test (applied for more than 2 subgroups). (Table.S30)

**Table.S7 Subgroup detailed information for Male/Female**

| **Group** | **16S Data** | **Total no.** | **Sample number in Table. S3** |
| --- | --- | --- | --- |
| Male | Cancer | 16 | F1, F2, F3, F4, F5, F8, F9, F11, F13, F17, F18, S1, S5, S6, S10, S11 |
|  | Normal | 20 | F1,F2,F3,F4,F5,F8,F9,F10,F11,F13,F17,F18,S2,S5,S6,S7,S10,S11,S13,S15 |
| Female | Cancer | 9 | F6, F14, F16, S3, S4, S8, S9, S12, S14 |
|  | Normal | 10 | F6, F14, F15, F16, S3, S4, S8, S9, S12, S14, |

In these 4 subgroups, separated cancer/normal 16S data were used, each group had around 10 subjects or more. The α-diversity indices based on species level were calculated by Mothur (v1.31.2), which included: observed species, chao, ace, shannon and simpson index. β-diversity analysis based on species level was done by QIIME (v1.80), and represented by unweighted_unifrac PCoA plots. (Figures for α-diversity and β-diversity were in Fig.2). Significantly different microbes among subgroups were screened out by kruskal.test (applied for more than 2 subgroups). (Table.S31)

**Table.S8 Subgroup detailed information for Smoker/Non-smoker**

| **Group** | **16S Data** | **Total no.** | **Sample number in Table. S3** |
| --- | --- | --- | --- |
| Smoker | Cancer | 10 | F3, F4, F5, F9, F11, F13, F17, F18, S6, S10 |
|  | Normal | 14 | F3, F4, F5, F9, F10, F11, F13, F17, F18, S2, S6, S7, S10, S13 |
| Non- | Cancer | 15 | F1, F2, F6, F8, F14, F16, S1, S3, S4, S5, S8, S9, S11, S12, S14 |
| Smoker | Normal | 16 | F1, F2, F6, F8, F14, F15, F16, S3, S4, S5, S8, S9, S11, S12, S14, S15 |

In these 4 subgroups, separated cancer/normal 16S data were used, each group had around 10 subjects or more. The α-diversity indices based on species level were calculated by Mothur (v1.31.2), which included: observed species, chao, ace, shannon and simpson index. β-diversity analysis based on species level was done by QIIME (v1.80), and represented by unweighted_unifrac PCoA plots. (Figures for α-diversity and β-diversity were in Fig.2). Significantly different microbes among subgroups were screened out by kruskal.test (applied for more than 2 subgroups). (Table.S32)

**The combined 16S normal/cancer data for 31 patients (24 paired subjects, unpaired sample: 1 cancer, 6 normal)**

**Table.S9 Subgroup detailed information for FLC/Sporadic, based on combined 16S data**

| **Group** | **16S Data** | **Total no.** | **Sample number in Table. S3** |
| --- | --- | --- | --- |
| FLC | combined | 16 | F1, F2, F3, F4, F5, F6, F8, F9, F10, F11, F13, F14, F15, F16, F17, F18 |
| Sporadic | combined | 15 | S1, S2, S3, S4, S5, S6, S7, S8, S9, S10, S11, S12, S13, S14, S15 |
| Benign | normal | 5 | B1, B2, B3, B4, B5 |

In these subgroups, combined cancer/normal 16S data were used, the benign group used the normal 16S data. Significantly different microbes among subgroups were screened out by PERMANOVA in R studio (R v4.1.1), only based on FLC/Sporadic, (Table.S29). PLSDA analysis was done in R studio (R v4.1.1), using 3 subgroups (Fig.1D).

**Table.S10 Subgroup detailed information for High-IAP/Low-IAP, based on combined 16S data**

| **Group** | **16S Data** | **Total no.** | **Sample number in Table. S3** |
| --- | --- | --- | --- |
| High-IAP | combined | 14 | F1, F2, F3, F4, F5, F8, F9, F11, F14, F15, F16, F17, S5, S6 |
| Low-IAP | combined | 17 | F6, F10, F13, F18, S1, S2, S3, S4, S7, S8, S9, S10, S11, S12, S13, S14, S15 |

In these subgroups, combined cancer/normal 16S data were used. PLSDA analysis was done in R studio (R v4.1.1), (Fig.1E).

**Table.S11 Subgroup detailed information for Male/Female, based on combined 16S data**

| **Group** | **16S Data** | **Total no.** | **Sample number in Table. S3** |
| --- | --- | --- | --- |
| Male | combined | 21 | F1, F2, F3, F4, F5, F8, F9, F10, F11, F13, F17, F18, S1, S2, S5, S6, S7, S10, S11, S13, S15 |
| Female | combined | 10 | F6, F14, F15, F16, S3, S4, S8, S9, S12, S14 |

In these subgroups, combined cancer/normal 16S data were used. PLSDA analysis was done in R studio (R v4.1.1), (Fig.1F).

**Table.S12 Subgroup detailed information for Smoker/Nonsmoker, based on combined 16S data**

| **Group** | **16S Data** | **Total no.** | **Sample number in Table. S3** |
| --- | --- | --- | --- |
| Smoker | combined | 14 | F3, F4, F5, F9, F10, F11, F13, F17, F18, S2, S6, S7, S10, S13 |
| Non-smoker | combined | 17 | F1, F2, F6, F8, F14, F15, F16, S1, S3, S4, S5, S8, S9, S11, S12, S14, S15 |

In these subgroups, combined cancer/normal 16S data were used. PLSDA analysis was done in R studio (R v4.1.1), (Fig.1G).

**Table.S13 Subgroup detailed information for patient age, based on combined 16S data**

| **Group Age** | **16S Data** | **Total no.** | **Sample number in Table. S3** |
| --- | --- | --- | --- |
| Age < 50 | combined | 9 | F2, F3, F5, F6, F8, F10, F11, F15, F18 |
| Age ≥ 50 | combined | 22 | F1, F4, F9, F13, F14, F16, F17, S1, S2, S3, S4, S5, S6, S7, S8, S9, S10, S11, S12, S13, S14, S15 |

In these subgroups, combined cancer/normal 16S data were used. Significantly different microbes among subgroups were screened out by wilcox.test (applied for 2 subgroups) in R studio (R v4.1.1), (Table.S33). PLSDA analysis was done in R studio (R v4.1.1), (Fig.3A).

**Table.S14 Subgroup detailed information for patient anatomy site, based on combined 16S data**

| **Group Anatomy** | **16S Data** | **Total no.** | **Sample number in Table. S3** |
| --- | --- | --- | --- |
| Left lung upper | combined | 7 | F3, F11, S2, S8, S9, S11, S14 |
| Left lung lower | combined | 7 | F1, F4, F6, F9, F10, F15, F18 |
| Right lung upper | combined | 8 | F2, F5, F8, S1, S5, S10, S12, S13 |
| Right lung lower | combined | 7 | F13, F14, F16, F17, S4, S6, S7 |
| Others | combined | 2 | S3, S15 |

In these subgroups, combined cancer/normal 16S data were used. Significantly different microbes were screened out by kruskal.test (applied for more than 2 subgroups) in R studio (R v4.1.1), (Table.S34), “others” were not included. PLSDA analysis was done in R studio (R v4.1.1), (Fig.3B).

**Table.S15 Subgroup detailed information for patient blood type, based on combined 16S data**

| **Group Blood type** | **16S Data** | **Total no.** | **Sample number in Table. S3** |
| --- | --- | --- | --- |
| A | combined | 9 | F3, F5, F15, F17, S2, S5, S10, S12, S15 |
| AB | combined | 3 | F6, F11, S4 |
| B | combined | 6 | F13, F18, S1, S6, S7, S9 |
| O | combined | 13 | F1, F2, F4, F8, F9, F10, F14, F16, S3, S8, S11, S13, S14 |

In these subgroups, combined cancer/normal 16S data were used. Significantly different microbes among subgroups were screened out by kruskal.test (applied for more than 2 subgroups) in R studio (R v4.1.1), (Table.S35). PLSDA analysis was done in R studio (R v4.1.1), (Fig.3C).

**Table.S16 Subgroup detailed information for patient histology, mainly based on combined 16S data**

| **Group Histology** | **16S Data** | **Total no.** | **Sample number in Table. S3** |
| --- | --- | --- | --- |
| AD | combined | 20 | F1, F2, F3, F5, F6, F8, F9, F13, F14, F15, F16, F17, S3, S4, S6, S8, S9, S10, S12, S14 |
| SCC | combined | 8 | F10, F18, S2, S5, S7, S11, S13, S15 |
| SCLC + others | combined | 3 | F4, F11, S1 |
| Benign | normal | 5 | B1, B2, B3, B4, B5 |

In these subgroups, combined cancer/normal 16S data were used, benign group used the normal tissue 16S data. Significantly different microbes among subgroups were screened out by wilcox.test (applied for 2 subgroups) in R studio (R v4.1.1), only AD/SCC were included (Table.S36). PLSDA analysis was done in R studio (R v4.1.1), (Fig.3D)

**Table.S17 Subgroup detailed information for patient tumor T stage, based on combined 16S data**

| **Group T stage** | **16S Data** | **Total no.** | **Sample number in Table. S3** |
| --- | --- | --- | --- |
| T1-2 | combined | 17 | F1, F2, F4, F5, F8, F9, F11, F18, S1, S3, S5, S6, S10, S11, S13, S14, S15 |
| T3-4 | combined | 14 | F3, F6, F10, F13, F14, F15, F16, F17, S2, S4, S7, S8, S9, S12 |

In these subgroups, combined cancer/normal 16S data were used. Significantly different microbes among subgroups were screened out by wilcox.test (applied for 2 subgroups) in R studio (R v4.1.1) (Table.S37). PLSDA analysis was done in R studio (R v4.1.1), (Fig.3E)

**Table.S18 Subgroup detailed information for patient tumor N stage, based on combined 16S data**

| **Group N stage** | **16S Data** | **Total no.** | **Sample number in Table. S3** |
| --- | --- | --- | --- |
| N0 | combined | 17 | F1, F2, F5, F6, F8, F9, F10, F16, F17, S1, S3, S5, S6, S7, S11, S13, S14 |
| N1-2 | combined | 14 | F3, F4, F11, F13, F14, F15, F18, S2, S4, S8, S9, S10, S12, S15 |

In these subgroups, combined cancer/normal 16S data were used. Significantly different microbes among subgroups were screened out by wilcox.test (applied for 2 subgroups) in R studio (R v4.1.1), (Table.S38). PLSDA analysis was done in R studio (R v4.1.1), (Fig.3F)

**Table.S19 Subgroup detailed information for patient tumor M stage, based on combined 16S data**

| **Group M stage** | **16S Data** | **Total no.** | **Sample number in Table. S3** |
| --- | --- | --- | --- |
| M0 | combined | 29 | F1, F2, F3, F4, F5, F6, F8, F9, F10, F11, F13, F14, F15, F16, F17, F18, S1, S2, S3, S4, S5, S6, S7, S8, S10, S11, S13, S14, S15 |
| M1 | combined | 2 | S9, S12 |

In these subgroups, combined cancer/normal 16S data were used. Significantly different microbes were screened by wilcox.test (applied for 2 subgroups) in R studio (R v4.1.1); but for M1 only had 2 patients, made it less conclusive, so we didn’t include this data in the tables. PLSDA analysis was done in R studio (R v4.1.1), (Fig.3G).

**Table.S20 Subgroup detailed information for patient tumor stage, based on combined 16S data**

| **Group stage** | **16S Data** | **Total no.** | **Sample number in Table. S3** |
| --- | --- | --- | --- |
| I-II | combined | 14 | F1, F2, F5, F6, F8, F9, F18, S1, S3, S5, S6, S11, S13, S14 |
| III-IV | combined | 17 | F3, F4, F10, F11, F13, F14, F15, F16, F17, S2, S4, S7, S8, S9, S10, S12, S15 |

In these subgroups, combined cancer/normal 16S data were used. Significantly different microbes among subgroups were screened out by wilcox.test (applied for 2 subgroups) in R studio (R v4.1.1), (Table.S39). PLSDA analysis was done in R studio (R v4.1.1), (Fig.3H).

**RNA-seq 29 patients: normal lung tissue**

**Table.S21 Subgroup detailed information for RNA-seq patient, FLC vs Sporadic**

| **Group** | **Tissue type** | **Total no.** | **Sample number in Table. S3** |
| --- | --- | --- | --- |
| FLC | normal | 18 | F1, F2, F3, F4, F5, F6, F7, F8, F9, F10, F11, F12, F13, F14, F16, F17, F18, F19 |
| Sporadic | normal | 11 | S2, S3, S4, S5, S7, S8, S9, S10, S12, S14, S15 |

Differentially expression genes (DEG) between subgroups were identified by DEGseq algorithms, (Table.S40).

**Table.S22 Subgroup detailed information for RNA-seq patient, High-IAP vs Low-IAP**

| **Group** | **Tissue type** | **Total no.** | **Sample number in Table. S3** |
| --- | --- | --- | --- |
| High-IAP | normal | 15 | F1, F2, F3, F4, F5, F7, F8, F9, F11, F12, F14, F16, F17, F19, S5 |
| Low-IAP | normal | 14 | F6, F10, F13, F18, S2, S3, S4, S7, S8, S9, S10, S12, S14, S15 |

Differentially expression genes (DEG) between subgroups were identified by DEGseq algorithms, (Table.S41).

**Table.S23 Subgroup detailed information for RNA-seq patient, Male vs Female**

| **Group** | **Tissue type** | **Total no.** | **Sample number in Table. S3** |
| --- | --- | --- | --- |
| Male | normal | 18 | F1, F2, F3, F4, F5, F8, F9, F10, F11, F13, F17, F18, F19, S2, S5, S7, S10, S15 |
| Female | normal | 11 | F6, F7, F12, F14, F16, S3, S4, S8, S9, S12, S14 |

Differentially expression genes (DEG) between subgroups were identified by DEGseq algorithms, (Table.S42).

**RESULTS**

**The α-diversity data for 4 groups**: FLC vs Sporadic, High-IAP vs Low-IAP, Female vs Male, Ever smoker vs Never smoker

Table.S24-S27 provided the α-diversity data for 4 groups: FLC vs Sporadic, High-IAP vs Low-IAP, Female vs Male, Ever smoker vs Never smoker. The α-diversity indices were calculated by Mothur (v1.31.2), suing separated normal/cancer tissue data. Since all the samples were lung tissues, all of them had the same dominate phylum, genera and species, that represented the basic microbiome of the organ lung. As a result, the α-diversity based on all species didn’t reach statistical significance. Although not significant, difference could be found among subgroups, these differences are mainly caused by subgroup-specific microbes in relatively low abundance, even not the dominate ones, these microbes could still have their influence on lung microenvironment.

**Table.S24 The α-diversity data for** **FLC vs Sporadic lung cancer (Fig2.A)**

| **α-diversity** | **Mean value and standard deviation** | | | | | | | | | | |
| --- | --- | --- | --- | --- | --- | --- | --- | --- | --- | --- | --- |
| Index | B_mean | (SD) | C1_mean | (SD) | C2_mean | (SD) | N1_mean | (SD) | N2_mean | (SD) | P.value ^a^ |
| Observed species | 325 | 26 | 313 | 24 | 333 | 34 | 318 | 33 | 315 | 36 | 0.73 |
| Chao | 351 | 35 | 335 | 31 | 353 | 34 | 339 | 39 | 335 | 41 | 0.74 |
| Ace | 349 | 32 | 335 | 32 | 355 | 34 | 341 | 39 | 336 | 40 | 0.78 |
| Shannon | 1.49 | 0.050 | 1.52 | 0.16 | 1.57 | 0.12 | 1.52 | 0.065 | 1.48 | 0.094 | 0.10 |
| Simpson | 0.364 | 0.013 | 0.359 | 0.033 | 0.351 | 0.021 | 0.353 | 0.013 | 0.367 | 0.020 | 0.10 |
| Coverage | 0.999 | 0.00025 | 0.999 | 0.00034 | 0.999 | 0.00025 | 0.999 | 0.00032 | 0.999 | 0.0003 | 0.89 |

a: P value calculated by Mothur (v1.31.2), P<0.05 for statistic significant;

B: Benign tumor adjacent normal tissue; C1/N1: cancer/normal tissue of familial lung cancer; C2/N2: cancer/normal tissue of sporadic lung cancer;

**Table.S25 The α-diversity data for** **High-IAP vs Low-IAP lung cancer (Fig2.B)**

| **α-diversity** | **Mean value and standard deviation** | | | | | | | | | | |
| --- | --- | --- | --- | --- | --- | --- | --- | --- | --- | --- | --- |
| Index | B_mean | (SD) | C.H_mean | (SD) | C.L_mean | (SD) | N.H_mean | (SD) | N.L_mean | (SD) | P.value ^a^ |
| Observed species | 325 | 26 | 315 | 26 | 330 | 33 | 324 | 33 | 311 | 34 | 0.73 |
| Chao | 351 | 35 | 336 | 30 | 351 | 36 | 345 | 36 | 331 | 41 | 0.69 |
| Ace | 349 | 32 | 336 | 30 | 352 | 37 | 346 | 36 | 332 | 42 | 0.81 |
| Shannon | 1.49 | 0.050 | 1.53 | 0.16 | 1.56 | 0.12 | 1.52 | 0.077 | 1.49 | 0.083 | 0.41 |
| Simpson | 0.364 | 0.013 | 0.357 | 0.034 | 0.353 | 0.022 | 0.355 | 0.015 | 0.364 | 0.019 | 0.49 |
| Coverage | 0.999 | 0.00025 | 0.999 | 0.00028 | 0.999 | 0.00032 | 0.999 | 0.00024 | 0.999 | 0.00036 | 0.86 |

a: P value calculated by Mothur (v1.31.2), P<0.05 for statistic significant;

B: Benign tumor adjacent normal tissue; C.H/N.H: cancer/normal tissue from high indoor air pollution region (High-IAP); C.L/N.L: cancer/normal tissue from low indoor air pollution region (Low-IAP);

**Table.S26 The α-diversity data for** **Female vs Male lung cancer (Fig2.C)**

| **α-diversity** | **Mean value and standard deviation** | | | | | | | | | | |
| --- | --- | --- | --- | --- | --- | --- | --- | --- | --- | --- | --- |
| Index | B_mean | (SD) | C.F_mean | (SD) | C.M_mean | (SD) | N.F_mean | (SD) | N.M_mean | (SD) | P.value ^a^ |
| Observed species | 325 | 26 | 330 | 22 | 318 | 33 | 314 | 44 | 318 | 30 | 0.83 |
| Chao | 351 | 35 | 346 | 28 | 342 | 36 | 330 | 49 | 341 | 34 | 0.90 |
| Ace | 349 | 32 | 347 | 29 | 342 | 37 | 331 | 50 | 342 | 35 | 0.97 |
| Shannon | 1.49 | 0.050 | 1.56 | 0.13 | 1.53 | 0.15 | 1.54 | 0.096 | 1.49 | 0.070 | 0.47 |
| Simpson | 0.364 | 0.013 | 0.355 | 0.025 | 0.355 | 0.031 | 0.351 | 0.021 | 0.363 | 0.015 | 0.65 |
| Coverage | 0.999 | 0.00025 | 0.999 | 0.00029 | 0.999 | 0.00027 | 0.999 | 0.00027 | 0.999 | 0.0003 | 0.15 |

a: P value calculated by Mothur (v1.31.2), P<0.05 for statistic significant;

B: Benign tumor adjacent normal tissue; C.F/N.F: cancer/normal tissue of female lung cancer; C.M/N.M: cancer/normal tissue of male lung cancer;

**Table.S27 The α-diversity data for** **Ever smoker vs Never smoker lung cancer (Fig2.D)**

| **α-diversity** | **Mean value and standard deviation** | | | | | | | | | | |
| --- | --- | --- | --- | --- | --- | --- | --- | --- | --- | --- | --- |
| Index | B_mean | (SD) | C.E_mean | (SD) | C.N_mean | (SD) | N.E_mean | (SD) | N.N_mean | (SD) | P.value ^a^ |
| Observed species | 325 | 26 | 321 | 30 | 323 | 31 | 320 | 28 | 314 | 39 | 0.98 |
| Chao | 351 | 35 | 346 | 34 | 341 | 33 | 343 | 33 | 333 | 44 | 0.84 |
| Ace | 349 | 32 | 345 | 36 | 342 | 34 | 345 | 33 | 333 | 44 | 0.94 |
| Shannon | 1.49 | 0.050 | 1.56 | 0.17 | 1.53 | 0.13 | 1.50 | 0.070 | 1.51 | 0.091 | 0.94 |
| Simpson | 0.364 | 0.013 | 0.348 | 0.033 | 0.360 | 0.025 | 0.360 | 0.015 | 0.359 | 0.020 | 0.88 |
| Coverage | 0.999 | 0.00025 | 0.999 | 0.00032 | 0.999 | 0.00027 | 0.999 | 0.00028 | 0.999 | 0.0003 | 0.25 |

a: P value calculated by Mothur (v1.31.2), P<0.05 for statistic significant;

B: Benign tumor adjacent normal tissue; C.E./N.E: cancer/normal tissue from ever smokers; C.N/N.N: cancer/normal tissue from never smokers;

**The β-diversity heatmap for 4 groups**: FLC vs Sporadic, High-IAP vs Low-IAP, Female vs Male, Ever smoker vs Never smoker

The β-diversity analysis was done by QIIME (v1.80), suing separated normal/cancer tissue data. The uniFrac is one of the β-diversity analysis method, which uses the system evolution information to compare the composition of community species between samples. Since different microbes were detected in low abundance, β-diversity was analyzed by unweighted_unifrac, which didn’t calculate the abundance of sequences. Because all the samples were lung tissues, all of them had the same dominate phylum, genera and species, that represented the basic microbiome of the organ lung. As a result, the β-diversity based on all species didn’t reach statistical significance, so there was no significant separation in the 4 heatmaps (Fig.S1). In order to dig out the small difference induced by those less abundant microbes, in the manuscript, we used unweighted_unifrac PCoA plots (Fig.2E-H), which looked for how much the two groups could overlap with each other, or how much they separated apart. In other words: the more overlap, the more similar of the 2 groups; the less overlap, the bigger difference, or bigger change. It was an alternative means to compare how similar of the 2 microbiomes, or how much they changed from normal to cancer.


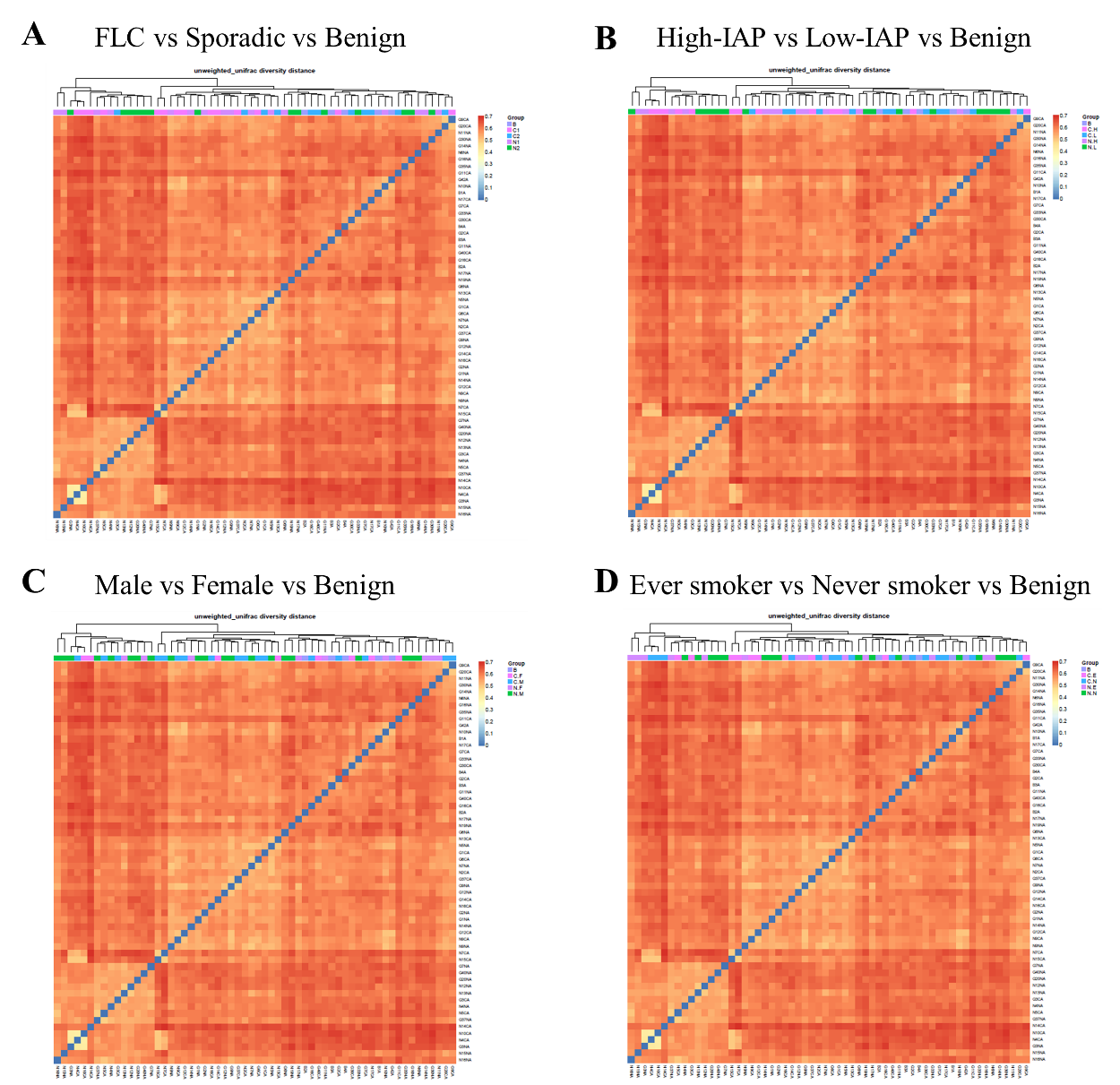


**Fig.S1 β-diversity heatmap by unweighted uniFrac, there was no statistic significant β-diversity among groups.**

A: FLC vs Sporadic vs Benign; B: High-IAP vs Low-IAP vs Benign;

C: Male vs Female vs Benign; D: Ever smoker vs Never smoker vs Benign.

Because different microbes existing in low abundance, β-diversity was analyzed by unweighted_unifrac, which didn’t calculate the abundance of microbe species. Since all the samples were lung tissues, all of them had the same dominate phylum, genera and species, that represented the basic microbiome of the organ lung. As a result, the β-diversity based on all species didn’t reach statistical significance, so there was also no significant separation in the 4 heatmaps.

**Analysis methods selection trials: PCA, LEFse and PLSDA**

The following part described why we chose PLSDA analysis to analyze our microbiome data, since PCA and LEFse analysis were also common methods for microbiome analysis. In fact, we indeed tried PCA and LEFse, but they seemed not working well on our microbiome data. Considering certain data structure should be matched with the right method to work properly. After a serious of trials, we found PLSDA really work good on our microbiome data.

**PCA and LEFse analysis**

For microbiome analysis, at the beginning, we do carried PCA and LEFse analysis for every subgroup, by using separate cancer/normal data. However PCA couldn’t separate subpopulations apart in all cases (Fig.S2), and LEFse provided deficient or no results (Fig.S3). That might be explained at least partly by our relatively small sample size and special subjects’ background, because FLC tissue samples were relatively rare. Actually, certain data structure should be matched with the right method to work properly. After a serious of trials, we found PLSDA indeed do better job; working for both combined and separate cancer/normal data (Fig.S4); PLSDA made clear separation in most subgroups; further, those separation could have meaningful explanations. Finally, we used PLSDA results in the manuscript.


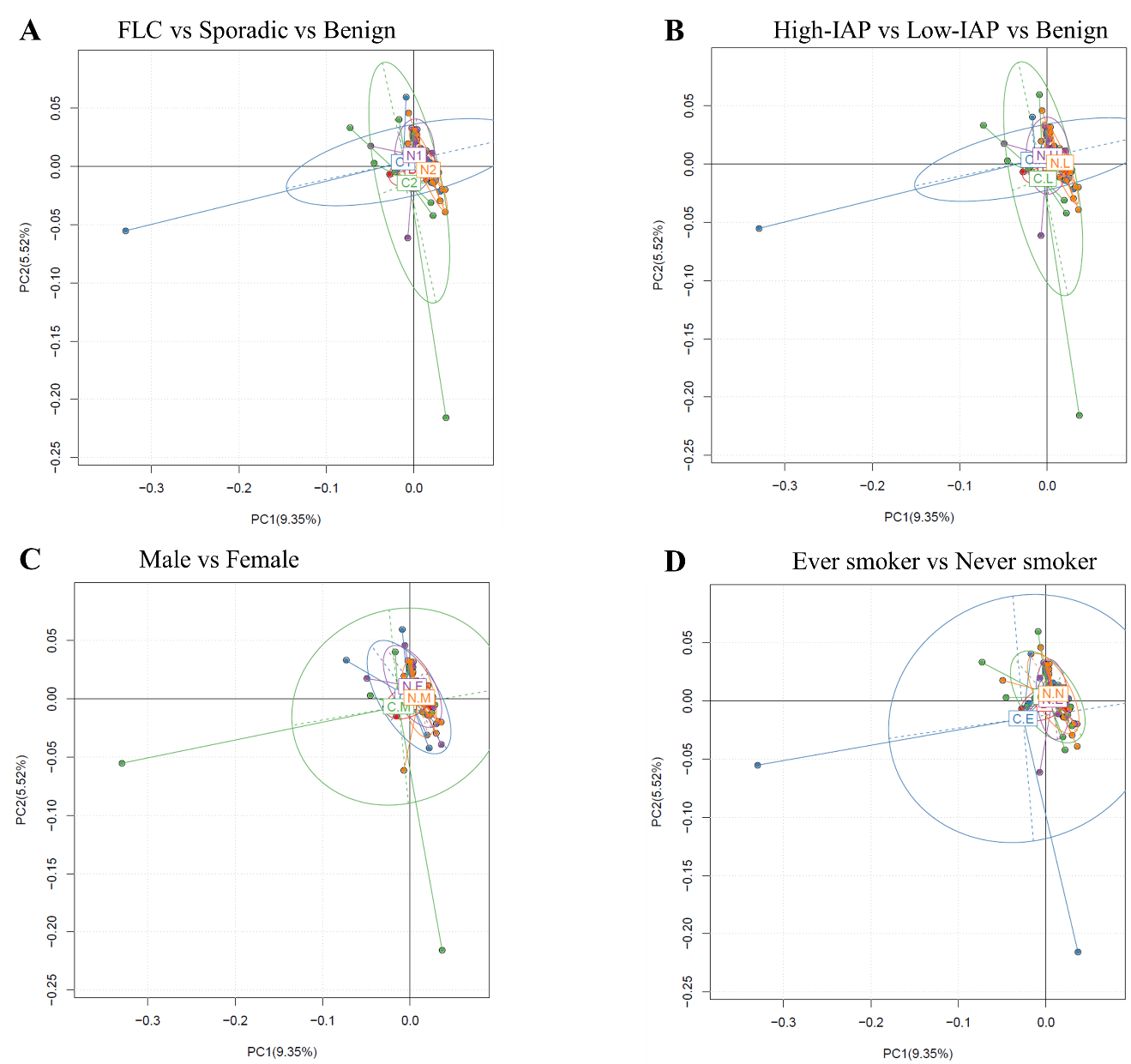


**Fig.S2 PCA analysis couldn’t separate subgroups apart in all cases.**

A: FLC vs Sporadic vs Benign; B: High-IAP vs Low-IAP vs Benign;

C: Male vs Female; D: Ever smoker vs Never smoker.

For microbiome analysis, PCA analysis were carried for different subgroup-division, by using separate cancer/normal data. Covering: FLC vs Sporadic, High-IAP vs Low-IAP, Female vs Male, Ever smoker vs Never smoker. However, PCA couldn’t separate subpopulations apart in all cases: all the subgroups almost crowded overlapped with each other.


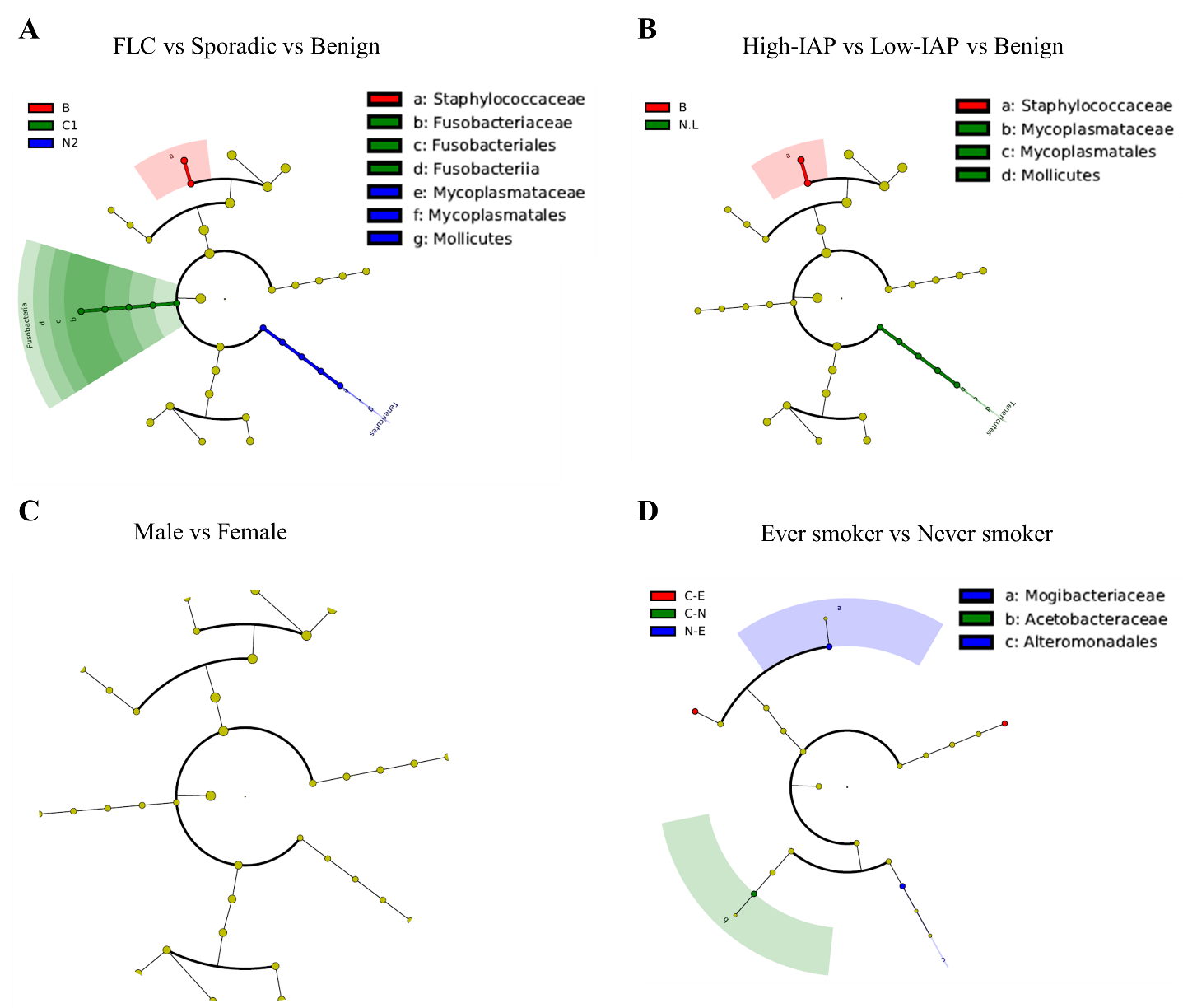


**Fig.S3** **LEFse analysis provided deficient or no results.**

A: FLC vs Sporadic vs Benign; B: High-IAP vs Low-IAP vs Benign;

C: Male vs Female; D: Ever smoker vs Never smoker.

For microbiome analysis, LEFse analysis were also carried for different subgroup-division, by using separate cancer/normal data. Covering: FLC vs Sporadic, High-IAP vs Low-IAP, Female vs Male, Ever smoker vs Never smoker. However, in LEFse analysis, many subgroups didn’t have any microbes selected out, especially in gender group, no different microbes were screen out. For those had some microbes, they were quite limited and lacking the diversity in a microbiome. As a result, we didn’t use LEFse for later analysis.


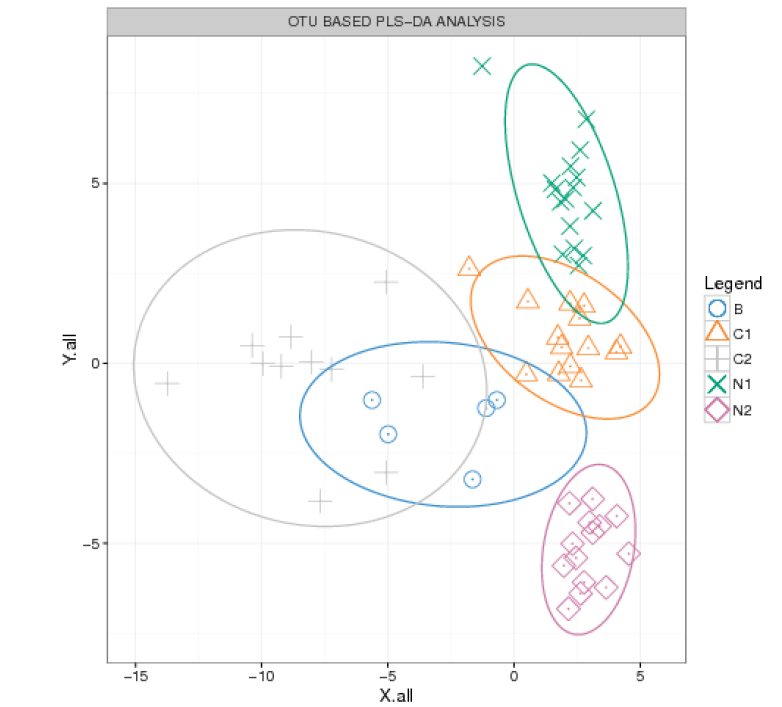


**Fig.S4 PLSDA analysis also worked for separate cancer/normal data.**

C1/N1: cancer/normal tissue of familial lung cancer; C2/N2: cancer/normal tissue of sporadic lung cancer; B: Benign tumor adjacent normal tissue.

For microbiome analysis, PLSDA analysis were also carried for FLC vs Sporadic, by using separate cancer/normal data. The results showed: C2 group were clearly separated from N2 group, with a distance in between, which indicated sporadic patients had bigger microbiome change from normal to cancer. On the other side, C1 group had small overlap whit N1 group, that meant FLC microbiome changed less from normal to cancer, which correctly matched to what we found for FLC microbiome: “less tend to change”, based on α-diversity and β-diversity in the manuscript. Since certain parameter had more than two subcategories (like blood type and TNM stage), and 16SrRNA data were not strictly paired, separate cancer/normal data would make very small subgroups, which might be too small for the algorithm. As a result, in the later analysis, we mainly use combined 16S cancer/normal data for PLSDA analysis.

**The benign group**

We used 5 benign tumors’ adjacent normal tissue as control. Actually, they indeed help in comparing subgroup-specific OTU in normal tissues; especially (Fig.1M, O), nonsmokers and low-IAP group both showed higher normal specific OTU than smokers and high-IAP group (504 vs 394, 500 vs 435); supporting smoking and IAP decreased specific-OTU biodiversity. Further, the benign group could be successfully separated from FLC and sporadic group by PLSDA using combined data (Fig.1D); but when using separate cancer/normal data, it had some overlap with sporadic caner (Fig.S4). That might partly be caused by our small number of benign cases, and too many small groups also made PLSDA analysis difficult to accurate. Finally, we used combined cancer/normal data for all PLSDA analysis in the manuscript.

In α-diversity, the benign group diversity were most similar to the sporadic normal and never smoker’s normal microbiome (Fig,2A,D); even not strictly normal, at least, benign group was more near to the less diseased microenvironment. But for its small number, we didn’t use it in all PLSDA analysis, and also not presented it in the β-diversity unweighted_unifrac PCoA plots (Fig.2E-H).

**References**

1. Barone-Adesi F, Chapman RS, Silverman DT, et al. Risk of lung cancer associated with domestic use of coal in Xuanwei, China: retrospective cohort study. BMJ 2012;345.

2. Chapman RS, Mumford JL, Harris DB, et al. The epidemiology of lung cancer in Xuan Wei, China: current progress, issues, and research strategies. Arch Environ Health 1988;43:180–185.

3. Mumford JL, Chapman RS, Harris DB, et al. Indoor air exposure to coal and wood combustion emissions associated with a high lung cancer rate in Xuan Wei, China. Environment International 1989;15:315–320.

4. Mumford JL, He XZ, Chapman RS, et al. Lung cancer and indoor air pollution in Xuan Wei, China. Science 1987;235:217–220.

5. Ding X, Chen Y, Yang J, et al. Characteristics of Familial Lung Cancer in Yunnan-Guizhou Plateau of China. Frontiers in oncology. 2018;8:637.

6. Chen Y, Huang Y, Kanwal M, et al. MUC16 in non-small cell lung cancer patients affected by familial lung cancer and indoor air pollution: clinical characteristics and cell behaviors. Translational lung cancer research. 2019;8(4):476–88.
