# Supplementary material for: A Multi-Omics Study of Familial Lung Cancer: Microbiome and Host Gene Expression Patterns
Source: Front Immunol. 2022 Apr 11;13:827953. doi: 10.3389/fimmu.2022.827953 (PMC9037597; doi:10.3389/fimmu.2022.827953)
Supplement: Supplementary Tables 1–3 — for clinicopathological characteristics of lung cancer patients; [file DataSheet_2.docx]

**Table.S1-S3 for clinicalpathological characteristics of lung cancer patients between groups.**

**Table S1. Clinicalpathological characteristics of 34 lung cancer patients divided by gender**

| **Variables** | **Total** | **Patient gender** | | ***P* value ^a^** |
| --- | --- | --- | --- | --- |
|  |  | **Male** | **Female** |  |
| Total number of patients | 34 | 22 (64.7%) | 12 (35.3%) |  |
| Average age: 56 years ( range 27 - 70 ) |  |  |  | 0.43 |
| ≧50 years | 24 (70.6%) | 14 (63.6%) | 10 (83.3%) |  |
| <50 years | 10 (29.4%) | 8 (36.4%) | 2 (16.7%) |  |
| Average height: 161 cm ( range 97 - 176 ) |  |  |  | 0.001 |
| ≧161 cm | 22 (64.7%) | 19 (86.4%) | 3 (25.0%) |  |
| <161 cm | 12 (35.3%) | 3 (13.6%) | 9 (75.0%) |  |
| Average weight: 62 kg ( range 42 - 80 ) |  |  |  | 0.29 |
| ≧62 kg | 19 (55.9%) | 14 (63.6%) | 5 (41.7%) |  |
| <62 kg | 15 (44.1%) | 8 (36.4%) | 7 (58.3%) |  |
| Blood type |  |  |  | 0.40 |
| A | 9 (26.5%) | 7 (31.8%) | 2 (16.7%) |  |
| B | 6 (17.6%) | 5 (22.7%) | 1 (8.3%) |  |
| AB | 4 (11.8%) | 2 (9.1%) | 2 (16.7%) |  |
| O | 15 (44.1%) | 8 (36.4%) | 7 (58.3%) |  |
| Smoking history |  |  |  | 0.00014 |
| Yes (Current or Ex-smoker) | 15 (44.1%) | 15 (68.2%) | 0 (0.0%) |  |
| Never | 19 (55.9%) | 7 (31.8%) | 12 (100.0%) |  |
| Familial lung cancer (FLC) |  |  |  | 0.72 |
| Positive | 19 (55.9%) | 13 (59.1%) | 6 (50.0%) |  |
| Negative | 15 (44.1%) | 9 (40.9%) | 6 (50.0%) |  |
| Indoor air pollution (IAP) |  |  |  | 0.72 |
| High | 17 (50.0%) | 12 (54.5%) | 5 (41.7%) |  |
| Low | 17 (50.0%) | 10 (45.5%) | 7 (58.3%) |  |
| Anatomy site |  |  |  | 0.49^b^ |
| Left lung | 15 (44.1%) | 9 (40.9%) | 6 (50.0%) |  |
| Right lung | 18 (52.9%) | 13 (59.1%) | 5 (41.7%) |  |
| Bilateral lung | 1 (3.0%) | 0 (0.0%) | 1 (8.3%) |  |
| Histology type |  |  |  | 0.0005^c^ |
| Adenocarcinoma (AD) | 22 (64.7%) | 10 (45.5%) | 12 (100.0%) |  |
| Squamous cell carcinoma (SCC) | 9 (26.5%) | 9 (40.9%) | 0 (0.0%) |  |
| Small cell lung cancer | 1 (3.0%) | 1 (4.5%) | 0 (0.0%) |  |
| Others | 2 (5.8%) | 2 (9.1%) | 0 (0.0%) |  |
| Stage |  |  |  | 0.018 |
| I | 11 (32.4%) | 9 (40.9%) | 2 (16.7%) |  |
| II | 3 (8.8%) | 2 (9.1%) | 1 (8.3%) |  |
| III | 16 (47.0%) | 11 (50.0%) | 5 (41.7%) |  |
| IV | 4 (11.8%) | 0 (0.0%) | 4 (33.3%) |  |
| Distant organ metastasis |  |  |  | 0.011 |
| Present | 4 (11.8%) | 0 (0.0%) | 4 (33.3%) |  |
| Absent | 30 (88.2%) | 22 (100.0%) | 8 (66.7%) |  |

^a^ For categorical variables, using Fisher’s exact test (2-tailed).

^b^ *p* value calculated for left/right lung only, other sites are not included.

^c^ *p* value calculated for AD/SCC only, other types are not included.

**Table S2. Clinicalpathological characteristics of 34 lung cancer patients divided by smoking**

| **Variables** | **Total** | **Smoking history** | | ***P* value ^a^** |
| --- | --- | --- | --- | --- |
|  |  | **Positive** | **Negative** |  |
| Total number of patients | 34 | 15 (44.1%) | 19 (55.9%) |  |
| Gender |  |  |  | 0.00014 |
| Male | 22 (64.7%) | 15 (100.0%) | 7 (36.8%) |  |
| Female | 12 (35.3%) | 0 (0.0%) | 12 (63.2%) |  |
| Average age: 56 years ( range 27 - 70 ) |  |  |  | 0.28 |
| ≧50 years | 24 (70.6%) | 9 (60.0%) | 15 (78.9%) |  |
| <50 years | 10 (29.4%) | 6 (40.0%) | 4 (21.1%) |  |
| Average height: 161 cm ( range 97 - 176 ) |  |  |  | 0.15 |
| ≧161 cm | 22 (64.7%) | 12 (80.0%) | 10 (52.6%) |  |
| <161 cm | 12 (35.3%) | 3 (20.0%) | 9 (47.4%) |  |
| Average weight: 62 kg ( range 42 - 80 ) |  |  |  | 0.31 |
| ≧62 kg | 19 (55.9%) | 10 (66.7%) | 9 (47.4%) |  |
| <62 kg | 15 (44.1%) | 5 (33.3%) | 10 (52.6%) |  |
| Blood type |  |  |  | 0.29 |
| A | 9 (26.5%) | 5 (33.3%) | 4 (21.1%) |  |
| B | 6 (17.6%) | 4 (26.7%) | 2 (10.5%) |  |
| AB | 4 (11.8%) | 2 (13.3%) | 2 (10.5%) |  |
| O | 15 (44.1%) | 4 (26.7%) | 11 (57.9%) |  |
| Familial lung cancer (FLC) |  |  |  | 0.31 |
| Positive | 19 (55.9%) | 10 (66.7%) | 9 (47.4%) |  |
| Negative | 15 (44.1%) | 5 (33.3%) | 10 (52.6%) |  |
| Indoor air pollution (IAP) |  |  |  | 1.0 |
| High | 17 (50.0%) | 8 (53.3%) | 9 (47.4%) |  |
| Low | 17 (50.0%) | 7 (46.7%) | 10 (52.6%) |  |
| Anatomy site |  |  |  | 1.0^b^ |
| Left lung | 15 (44.1%) | 7 (46.7%) | 8 (42.1%) |  |
| Right lung | 18 (52.9%) | 8 (53.3%) | 10 (52.6%) |  |
| Bilateral lung | 1 (3.0%) | 0 (0.0%) | 1 (5.3%) |  |
| Histology type |  |  |  | 0.11^c^ |
| Adenocarcinoma (AD) | 22 (64.7%) | 7 (46.7%) | 15 (78.9%) |  |
| Squamous cell carcinoma (SCC) | 9 (26.5%) | 6 (40.0%) | 3 (15.8%) |  |
| Small cell lung cancer | 1 (3.0%) | 1 (6.7%) | 0 (0.0%) |  |
| Others | 2 (5.8%) | 1 (6.7%) | 1 (5.3%) |  |
| Stage |  |  |  | 0.06 |
| I | 11 (32.4%) | 4 (26.7%) | 7 (36.8%) |  |
| II | 3 (8.8%) | 1 (6.7%) | 2 (10.5%) |  |
| III | 16 (47.0%) | 10 (66.7%) | 6 (31.6%) |  |
| IV | 4 (11.8%) | 0 (0.0%) | 4 (21.1%) |  |
| Distant organ metastasis |  |  |  | 0.11 |
| Present | 4 (11.8%) | 0 (0.0%) | 4 (21.1%) |  |
| Absent | 30 (88.2%) | 15 (100.0%) | 15 (78.9%) |  |

^a^ For categorical variables, using Fisher’s exact test (2-tailed).

^b^ *p* value calculated for left/right lung only, other sites are not included.

^c^ *p* value calculated for AD/SCC only, other types are not included.

**Table.S3 Clinicalpathological characteristics of 34 lung cancer patients and 5 benign tumor cases**

| **No.** | **Variables** | | | | | | | | | | | | |  | **Sequencing type ^b^** | | |
| --- | --- | --- | --- | --- | --- | --- | --- | --- | --- | --- | --- | --- | --- | --- | --- | --- | --- |
|  | Sex | Age | Blood  type | Height  (cm) | Weight  (kg) | Smoke | Indoor air  pollution | Anatomy site | Histology type | T | N | M | Stage |  | 16S  Cancer | 16S  Normal | RNA  -seq |
| F1 | M | 52 | O | 165 | 60 | N | H | left lung (lower) | AD | T1 | N0 | M0 | I |  | + | + | + |
| F2 | M | 41 | O | 165 | 75 | N | H | right lung (upper) | AD | T2 | N0 | M0 | I |  | + | + | + |
| F3 | M | 38 | A | 150 | 54 | Y | H | left lung (upper) | AD | T4 | N2 | M0 | III |  | + | + | + |
| F4 | M | 54 | O | 163 | 66 | Y | H | left lung (lower) | SCLC | T2 | N2 | M0 | III |  | + | + | + |
| F5 | M | 49 | A | 171 | 77 | Y | H | right lung (upper) | AD | T2 | N0 | M0 | I |  | + | + | + |
| F6 | F | 27 | AB | 156 | 49 | N | L | left lung (lower) | AD | T3 | N0 | M0 | II |  | + | + | + |
| F7 | F | 57 | O | 157 | 55 | N | H | left lung (lower) | AD | T4 | N0 | M1 | IV |  | - | - | + |
| F8 | M | 47 | O | 168 | 64 | N | H | right lung (upper) | AD | T1 | N0 | M0 | I |  | + | + | + |
| F9 | M | 54 | O | 175 | 80 | Y | H | left lung (lower) | AD | T1 | N0 | M0 | I |  | + | + | + |
| F10 | M | 47 | O | 168 | 72 | Y | L | left lung (lower) | SCC | T4 | N0 | M0 | III |  | - | + | + |
| F11 | M | 49 | AB | 169 | 68 | Y | H | left lung (upper) | NA ^a^ | T2 | N2 | M0 | III |  | + | + | + |
| F12 | F | 56 | O | 147 | 65 | N | H | left and right lung | AD | T4 | N2 | M1 | IV |  | - | - | + |
| F13 | M | 57 | B | 165 | 60 | Y | L | right lung (lower) | AD | T4 | N2 | M0 | III |  | + | + | + |
| F14 | F | 55 | O | 162 | 62 | N | H | right lung (lower) | AD | T3 | N2 | M0 | III |  | + | + | + |
| F15 | F | 43 | A | 165 | 60 | N | H | left lung (lower) | AD | T4 | N2 | M0 | III |  | - | + | - |
| F16 | F | 51 | O | 97 | 65 | N | H | right lung (lower) | AD | T4 | N0 | M0 | III |  | + | + | + |
| F17 | M | 63 | A | 155 | 58 | Y | H | right lung (lower) | AD | T4 | N0 | M0 | III |  | + | + | + |
| F18 | M | 48 | B | 168 | 53 | Y | L | left lung (lower) | SCC | T2 | N1 | M0 | II |  | + | + | + |
| F19 | M | 40 | AB | 173 | 64 | Y | H | right lung (lower) | SCC | T3 | N1 | M0 | III |  | - | - | + |
| S1 | M | 60 | B | 175 | 75 | N | L | right lung (upper) | AD+SCC | T2 | N0 | M0 | II |  | + | - | - |
| S2 | M | 70 | A | 170 | 66 | Y | L | left lung (upper) | SCC | T3 | N2 | M0 | III |  | - | + | + |
| S3 | F | 52 | O | 150 | 62 | N | L | right lung (middle) | AD | T2 | N0 | M0 | I |  | + | + | + |
| S4 | F | 60 | AB | 149 | 42 | N | L | right lung (lower) | AD | T3 | N2 | M0 | III |  | + | + | + |
| S5 | M | 69 | A | 165 | 60 | N | H | right lung (upper) | SCC | T2 | N0 | M0 | I |  | + | + | + |
| S6 | M | 67 | B | 170 | 65 | Y | H | right lung (lower) | AD | T1 | N0 | M0 | I |  | + | + | - |
| S7 | M | 67 | B | 160 | 53 | Y | L | right lung (lower) | SCC | T4 | N0 | M0 | III |  | - | + | + |
| S8 | F | 66 | O | 150 | 50 | N | L | left lung (upper) | AD | T4 | N2 | M0 | III |  | + | + | + |
| S9 | F | 70 | B | 150 | 51 | N | L | left lung (upper) | AD | T4 | N2 | M1 | IV |  | + | + | + |
| S10 | M | 70 | A | 162 | 72 | Y | L | right lung (upper) | AD | T2 | N2 | M0 | III |  | + | + | + |
| S11 | M | 62 | O | 176 | 51 | N | L | left lung (upper) | SCC | T1 | N0 | M0 | I |  | + | + | - |
| S12 | F | 64 | A | 162 | 47 | N | L | right lung (upper) | AD | T4 | N2 | M1 | IV |  | + | + | + |
| S13 | M | 70 | O | 168 | 72 | Y | L | right lung (upper) | SCC | T2 | N0 | M0 | I |  | - | + | - |
| S14 | F | 64 | O | 158 | 64 | N | L | left lung (upper) | AD | T2 | N0 | M0 | I |  | + | + | + |
| S15 | M | 58 | A | 168 | 74 | N | L | right lung (whole) | SCC | T2 | N2 | M0 | III |  | - | + | + |
| B1 | M | 46 | AB | 170 | 60 | N | L | right lung (upper) | benign tumor |  |  |  |  |  | - | + | - |
| B2 | F | 60 | A | 170 | 60 | N | L | left lung (lower) | benign tumor |  |  |  |  |  | - | + | - |
| B3 | M | 60 | A | 168 | 65 | Y | L | right lung (upper) | benign tumor |  |  |  |  |  | - | + | - |
| B4 | F | 52 | AB | 171 | 60 | N | L | left lung (lower) | benign tumor |  |  |  |  |  | - | + | - |
| B5 | M | 75 | B | 162 | 66 | Y | L | left lung (upper) | benign tumor |  |  |  |  |  | - | + | - |

Abbreviation: F, familial lung cancer; S, sporadic lung cancer; B, benign tumor; M, male; F, female; Y, yes; N, no; H, high; L, low; AD, Adenocarcinoma; SCC, squamous cell carcinoma; SCLC, small cell lung cancer; +, sequenced; -, no sequenced;

a: The tumor showed extremely low differentiation, and can’t be classified as AD or SCC, indicating high degree of malignancy.

b: Because, familial lung cancer tissue samples were relatively rare, and not all the tissue parts had enough size for both 16S rRNA and RNA-seq, moreover, not every sample fit the strict quality control standard for sequencing. In order to analyze valuable samples as much as possible; firstly, we chose 24 paired tissue samples, plus unpaired sample: 1 cancer, 6 normal and 5 benign tumors (adjacent normal) for 16S rRNA. After that, 29 normal tissue samples from the same patient pool were selected for RNA-seq.
