# Supplementary material for: A Multi-Omics Study of Familial Lung Cancer: Microbiome and Host Gene Expression Patterns
Source: Front Immunol. 2022 Apr 11;13:827953. doi: 10.3389/fimmu.2022.827953 (PMC9037597; doi:10.3389/fimmu.2022.827953)
Supplement: Supplementary Tables 28–39 — for significantly different microbes between groups; [file DataSheet_3.docx]

**Table.S28-S39 for significantly different microbes between groups.**

**Table.S28 Significantly different microbes between** **familial and sporadic lung cancer. Identified at genus and species level**

| **Taxa** | **Mean relative abundance** | | | | | | | | **P.value ^a^** | **FDR** |
| --- | --- | --- | --- | --- | --- | --- | --- | --- | --- | --- |
| **Genus** | C1_mean | (SD) | C2_mean | (SD) | N1_mean | (SD) | N2_mean | (SD) |  |  |
| Capnocytophaga | 0.020 | (0.062) | 0.011 | (0.023) | 0.0042 | (0.00913) | 0 | (0) | 0.000061 | 0.0067 |
| Comamonas | 0.0052 | (0.0079) | 0.0024 | (0.0038) | 0.0057 | (0.00731) | 0.0068 | (0.0076) | 0.000071 | 0.0067 |
| Fusobacterium | 0.11 | (0.40) | 0.018 | (0.023) | 0.0026 | (0.00421) | 0.0078 | (0.012) | 0.0042 | 0.11 |
| Gemmata | 0.00034 | (0.0013) | 0 | (0) | 0.0013 | (0.00390) | 0 | (0) | 0.0089 | 0.17 |
| Lachnoanaerobaculum | 0.0013 | (0.0048) | 0.0050 | (0.010) | 0.00014 | (0.000571) | 0 | (0) | 0.000085 | 0.0067 |
| Marinococcus | 0.00095 | (0.0036) | 0.0033 | (0.0065) | 0 | 0 | 0 | (0) | 0.0046 | 0.11 |
| Mycoplasma | 0.0019 | (0.0059) | 0.048 | (0.11) | 0.0018 | (0.00604) | 0.10 | (0.18) | 0.00042 | 0.022 |
| Oligella | 0.010 | (0.0076) | 0.012 | (0.017) | 0.0063 | (0.0106) | 0.0074 | (0.0098) | 0.0037 | 0.10 |
| Peptococcus | 0 | (0) | 0 | (0) | 0.00015 | (0.000608) | 0.00064 | (0.0024) | 0.0025 | 0.083 |
| Rhodococcus | 0.0018 | (0.0039) | 0.0089 | (0.0094) | 0.0028 | (0.00442) | 0.0056 | (0.0087) | 0.00066 | 0.029 |
| Rubellimicrobium | 0.0074 | (0.0062) | 0.0076 | (0.012) | 0.0034 | (0.00779) | 0.0082 | (0.013) | 0.00094 | 0.037 |
| Sphingopyxis | 0.0021 | (0.0065) | 0.0034 | (0.0083) | 0.0015 | (0.00323) | 0.0029 | (0.0049) | 0.000025 | 0.0049 |
| Sphingomonas ^b^ | 0.049 | (0.083) | 0.044 | (0.028) | 0.038 | (0.0329) | 0.039 | (0.020) | 0.010 | 0.17 |
| Staphylococcus | 0.12 | (0.12) | 0.14 | (0.072) | 0.090 | (0.0436) | 0.092 | (0.065) | 0.00045 | 0.022 |
| **Species** |  |  |  |  |  |  |  |  |  |  |
| Capnocytophaga_ochracea | 0.017 | (0.056) | 0.0082 | (0.020) | 0.0031 | (0.0069) | 0 | (0) | 0.0041 | 0.13 |
| Lachnoanaerobaculum_orale | 0.0013 | (0.0048) | 0.0050 | (0.010) | 0.00014 | (0.00057) | 0 | (0) | 0.000085 | 0.0068 |
| Lactobacillus_helveticus | 0.030 | (0.015) | 0.021 | (0.020) | 0.036 | (0.020) | 0.034 | (0.017) | 0.048 | 0.30 |
| Lactobacillus_paralimentarius | 0.0027 | (0.0056) | 0.0014 | (0.0028) | 0.0010 | (0.0030) | 0.0073 | (0.0097) | 0.032 | 0.26 |
| Sphingomonas_wittichii | 0.0017 | (0.0047) | 0.0034 | (0.0058) | 0 | (0) | 0.00097 | (0.0026) | 0.018 | 0.20 |
| Sphingopyxis_alaskensis | 0.00095 | (0.0035) | 0.0024 | (0.0079) | 0.0015 | (0.0032) | 0.0029 | (0.0049) | 0.000001 | 0.00016 |

a: kruskal.test P<0.01 set for genus and P<0.05 for species, based on the availability and abundance of different microbes in each taxa. Analyzed independently, not every genus had species met P<0.05.

b: Sphingomonas is of particular interest to us, so we included it in each table to compare the abundance.

C1/N1: cancer/normal tissue of familial lung cancer; C2/N2: cancer/normal tissue of sporadic lung cancer;

**Table.S29 Significantly different microbes between familial and sporadic lung cancer. Identified by PERMANOVA**

| **Taxa** | **Mean relative abundance** | |  | **P.value ^a^** |  |  |
| --- | --- | --- | --- | --- | --- | --- |
| **Genus** | FLC_mean | Sporadic_mean |  |  | Log foldchange | Coverage |
| Staphylococcus | 2.3 | 5.9 |  | 0.0024 | -1.5 | 1.0 |
| Rubellimicrobium | 0.15 | 0 |  | 0.0074 | 4.1 | 0.47 |
| Alteromonadales;Other;Other ^b^ | 0.14 | 0 |  | 0.018 | 3.9 | 0.42 |
| Acetobacteraceae;Other ^b^ | 0.14 | 0 |  | 0.027 | 4.1 | 0.36 |
| Oligella | 0.18 | 0.029 |  | 0.039 | 2.6 | 0.61 |
| Comamonas | 0.083 | 0 |  | 0.044 | 3.2 | 0.42 |
| Sphingomonas | 0.91 | 0.40 |  | 0.045 | 1.12 | 1.0 |

a: Permutational multivariate analysis of variance (PERMANOVA), P<0.05 set for genus.

b: The exact genus of these taxa were not identified, so we included its order and family information.

FLC: familial lung cancer, Sporadic: sporadic lung cancer.

**Table.S30 significantly different microbes in lung cancer between** **high and low indoor air pollution region.**

| **Taxa** | **Mean relative abundance** | | | | | | | | **P.value ^a^** | **FDR** |
| --- | --- | --- | --- | --- | --- | --- | --- | --- | --- | --- |
| **Genus** | C.H_mean | (SD) | C.L_mean | (SD) | N.H_mean | (SD) | N.L_mean | (SD) |  |  |
| Acidovorax | 0.0036 | (0.0041) | 0.0098 | (0.0067) | 0.0048 | (0.0060) | 0.013 | (0.012) | 0.0022 | 0.081 |
| Actinomyces | 0.0080 | (0.018) | 0.037 | (0.047) | 0.015 | (0.032) | 0.0068 | (0.014) | 0.0097 | 0.14 |
| Butyricicoccus | 0.0013 | (0.0035) | 0 | (0) | 0.0037 | (0.0051) | 0.00088 | (0.0035) | 0.0093 | 0.14 |
| Capnocytophaga | 0.0033 | (0.0048) | 0.029 | (0.068) | 0.0031 | (0.0081) | 0.0015 | (0.0059) | 0.0001 | 0.0082 |
| Comamonas | 0.0031 | (0.0054) | 0.0049 | (0.0076) | 0.0058 | (0.0074) | 0.0065 | (0.0075) | 0.0001 | 0.0082 |
| Fusobacterium | 0.12 | (0.41) | 0.019 | (0.024) | 0.0051 | (0.0063) | 0.0050 | (0.011) | 0.0083 | 0.14 |
| Lachnoanaerobaculum | 0.0014 | (0.0050) | 0.0046 | (0.0096) | 0.00016 | (0.00061) | 0 | (0) | 0.0001 | 0.0082 |
| Marinococcus | 0.00090 | (0.0033) | 0.0031 | (0.0064) | 0 | (0) | 0 | (0) | 0.0055 | 0.11 |
| Oligella | 0.014 | (0.014) | 0.0074 | (0.0094) | 0.011 | (0.013) | 0.0033 | (0.0043) | 0.00071 | 0.035 |
| Peptococcus | 0 | (0) | 0 | (0) | 0.00064 | (0.0024) | 0.00015 | (0.00061) | 0.0025 | 0.083 |
| Rhodococcus | 0.0029 | (0.0065) | 0.0072 | (0.0084) | 0.0053 | (0.0089) | 0.0031 | (0.0042) | 0.0038 | 0.11 |
| Rubellimicrobium | 0.0071 | (0.0053) | 0.0080 | (0.012) | 0.0049 | (0.011) | 0.0063 | (0.011) | 0.0020 | 0.080 |
| Selenomonas | 0.012 | (0.035) | 0.022 | (0.041) | 0.0064 | (0.018) | 0.00015 | (0.00059) | 0.0074 | 0.13 |
| Sphingomonas ^b^ | 0.033 | (0.020) | 0.063 | (0.089) | 0.037 | (0.030) | 0.040 | (0.025) | 0.019 | 0.22 |
| Sphingopyxis | 0.00036 | (0.00087) | 0.0052 | (0.0099) | 0.0016 | (0.0037) | 0.0026 | (0.0044) | 0.000014 | 0.0028 |
| Staphylococcus | 0.13 | (0.14) | 0.12 | (0.041) | 0.085 | (0.045) | 0.096 | (0.061) | 0.00049 | 0.028 |
| Thermomonas | 0.0080 | (0.015) | 0.0020 | (0.0048) | 0.0041 | (0.0060) | 0 | (0) | 0.0074 | 0.13 |
| **Species** |  |  |  |  |  |  |  |  |  |  |
| Capnocytophaga_ochracea | 0.0023 | (0.0046) | 0.025 | (0.061) | 0.0018 | (0.0045) | 0.0015 | (0.0059) | 0.0065 | 0.16 |
| Lachnoanaerobaculum_orale | 0.0014 | (0.0050) | 0.0046 | (0.0096) | 0.00016 | (0.00061) | 0 | (0) | 0.0001 | 0.008 |
| Lactobacillus_helveticus | 0.032 | (0.015) | 0.019 | (0.017) | 0.039 | (0.016) | 0.031 | (0.019) | 0.0095 | 0.16 |
| Sphingomonas_wittichii | 0.0018 | (0.0049) | 0.0032 | (0.0056) | 0 | (0) | 0.00085 | (0.0025) | 0.031 | 0.23 |
| Sphingopyxis_alaskensis | 0 | (0) | 0.0033 | (0.0081) | 0.0016 | (0.0037) | 0.0026 | (0.0044) | 0.000001 | 0.00016 |

a: kruskal.test P<0.01 set for genus and P<0.05 for species, based on the availability and abundance of different microbes in each taxa. Analyzed independently, not every genus had species met P<0.05.

b: Sphingomonas is of particular interest to us, so we included it in each table to compare the abundance.

C.H/N.H: cancer/normal tissue from high indoor air pollution region (High-IAP); C.L/N.L: cancer/normal tissue from low indoor air pollution region (Low-IAP);

**Table.S31 Significantly different microbes between female and male lung cancer. Identified at genus and species level.**

| **Taxa** | **Mean relative abundance** | | | | | | | | **P.value ^a^** | **FDR** |
| --- | --- | --- | --- | --- | --- | --- | --- | --- | --- | --- |
| **Genus** | C.F_mean | (SD) | C.M_mean | (SD) | N.F_mean | (SD) | N.M_mean | (SD) |  |  |
| Capnocytophaga | 0.015 | (0.027) | 0.016 | (0.056) | 0.0059 | (0.012) | 0.00067 | (0.0021) | 0.000094 | 0.0093 |
| Comamonas | 0.0072 | (0.0095) | 0.0024 | (0.0040) | 0.0051 | (0.0067) | 0.0067 | (0.0077) | 0.000046 | 0.0061 |
| Lachnoanaerobaculum | 0.0031 | (0.0063) | 0.0028 | (0.0083) | 0.00025 | (0.00076) | 0 | (0) | 0.00013 | 0.010 |
| Marinococcus | 0.0024 | (0.0068) | 0.0018 | (0.0042) | 0 | (0) | 0 | (0) | 0.0099 | 0.23 |
| Oligella | 0.0057 | (0.0054) | 0.013 | (0.014) | 0.0079 | (0.012) | 0.0063 | (0.0094) | 0.0036 | 0.11 |
| Peptococcus | 0 | (0) | 0 | (0) | 0 | (0) | 0.00055 | (0.0020) | 0.0020 | 0.072 |
| Rheinheimera | 0.0054 | (0.0065) | 0.0019 | (0.0049) | 0 | (0) | 0.0034 | (0.0059) | 0.0012 | 0.055 |
| Rhodococcus | 0.0071 | (0.011) | 0.0039 | (0.0058) | 0.0013 | (0.0021) | 0.0053 | (0.0077) | 0.0074 | 0.20 |
| Rubellimicrobium | 0.014 | (0.013) | 0.0046 | (0.0051) | 0.0082 | (0.013) | 0.0046 | (0.0099) | 0.00054 | 0.030 |
| Sphingomonas ^b^ | 0.086 | (0.11) | 0.029 | (0.014) | 0.040 | (0.032) | 0.038 | (0.026) | 0.0099 | 0.23 |
| Sphingopyxis | 0.0014 | (0.0041) | 0.0032 | (0.0083) | 0 | (0) | 0.0031 | (0.0046) | 0.000004 | 0.00079 |
| Staphylococcus | 0.11 | (0.063) | 0.13 | (0.12) | 0.10 | (0.049) | 0.088 | (0.056) | 0.0010 | 0.050 |
| Vagococcus | 0.0044 | (0.0085) | 0.00026 | (0.00074) | 0 | (0) | 0 | (0) | 0.0020 | 0.072 |
| **Species** |  |  |  |  |  |  |  |  |  |  |
| Capnocytophaga_ochracea | 0.012 | (0.022) | 0.014 | (0.051) | 0.0044 | (0.0089) | 0.00045 | (0.0016) | 0.0058 | 0.14 |
| Lachnoanaerobaculum_orale | 0.0031 | (0.0063) | 0.0028 | (0.0083) | 0.00025 | (0.00076) | 0 | (0) | 0.00013 | 0.010 |
| Sphingomonas_wittichii | 0.0044 | (0.0066) | 0.0015 | (0.0043) | 0.0005 | (0.0015) | 0.00043 | (0.0020) | 0.037 | 0.26 |
| Sphingopyxis_alaskensis | 0 | (0) | 0.0023 | (0.0069) | 0 | (0) | 0.0031 | (0.0046) | 0 | 0 |

a: kruskal.test P<0.01 set for genus and P<0.05 for species, based on the availability and abundance of different microbes in each taxa. Analyzed independently, not every genus had species met P<0.05.

b: Sphingomonas is of particular interest to us, so we included it in each table to compare the abundance.

C.F/N.F: cancer/normal tissue of female lung cancer; C.M/N.M: cancer/normal tissue of male lung cancer.

**Table.S32 Significantly different microbes between ever and never smokers. Identified at genus and species level.**

| **Taxa** | **Mean relative abundance** | | | | | | | | **P.value ^a^** | **FDR** |
| --- | --- | --- | --- | --- | --- | --- | --- | --- | --- | --- |
| **Genus** | C.E_mean | (SD) | C.N_mean | (SD) | N.E_mean | (SD) | N.N_mean | (SD) |  |  |
| Capnocytophaga | 0.025 | (0.074) | 0.0094 | (0.020) | 0.0010 | (0.0026) | 0.0033 | (0.0092) | 0.00013 | 0.012 |
| Comamonas | 0.0025 | (0.0039) | 0.0049 | (0.0077) | 0.0051 | (0.0060) | 0.0072 | (0.0084) | 0.000094 | 0.012 |
| Lachnoanaerobaculum | 0.0018 | (0.0057) | 0.0037 | (0.0087) | 0 | (0) | 0.00014 | (0.00057) | 0.00015 | 0.012 |
| Marinococcus | 0.0013 | (0.0042) | 0.0024 | (0.0057) | 0 | (0) | 0 | (0) | 0.0086 | 0.19 |
| Oligella | 0.010 | (0.0082) | 0.012 | (0.014) | 0.0055 | (0.0072) | 0.0079 | (0.012) | 0.0063 | 0.15 |
| Peptococcus | 0 | (0) | 0 | (0) | 0.00017 | (0.00065) | 0.00056 | (0.0023) | 0.0025 | 0.11 |
| Rhodococcus | 0.0025 | (0.0045) | 0.0065 | (0.0090) | 0.0062 | (0.0089) | 0.0023 | (0.0036) | 0.0038 | 0.12 |
| Rubellimicrobium | 0.0037 | (0.0047) | 0.010 | (0.011) | 0.0023 | (0.0049) | 0.0086 | (0.013) | 0.00084 | 0.042 |
| Sphingomonas ^b^ | 0.030 | (0.018) | 0.058 | (0.080) | 0.041 | (0.028) | 0.036 | (0.027) | 0.016 | 0.24 |
| Sphingopyxis | 0.0029 | (0.0076) | 0.0025 | (0.0072) | 0.0028 | (0.0047) | 0.0015 | (0.0034) | 0.000024 | 0.0047 |
| Staphylococcus | 0.16 | (0.15) | 0.11 | (0.051) | 0.080 | (0.047) | 0.10 | (0.059) | 0.00086 | 0.042 |
| **Species** |  |  |  |  |  |  |  |  |  |  |
| Capnocytophaga_ochracea | 0.021 | (0.066) | 0.0075 | (0.017) | 0.00067 | (0.0019) | 0.0025 | (0.0069) | 0.0089 | 0.18 |
| Lachnoanaerobaculum_orale | 0.0018 | (0.0057) | 0.0037 | (0.0087) | 0 | (0) | 0.00014 | (0.00057) | 0.00015 | 0.012 |
| Lactobacillus_helveticus | 0.026 | (0.017) | 0.026 | (0.018) | 0.028 | (0.016) | 0.040 | (0.018) | 0.035 | 0.31 |
| Sphingomonas_wittichii | 0.00067 | (0.0021) | 0.0036 | (0.0063) | 0.00065 | (0.0024) | 0.00028 | (0.0011) | 0.025 | 0.27 |
| Sphingopyxis_alaskensis | 0.0013 | (0.0042) | 0.0017 | (0.0067) | 0.0028 | (0.0047) | 0.0015 | (0.0034) | 0.000001 | 0.00016 |

a: kruskal.test P<0.01 set for genus and P<0.05 for species, based on the availability and abundance of different microbes in each taxa. Analyzed independently, not every genus had species met P<0.05.

b: Sphingomonas is of particular interest to us, so we included it in each table to compare the abundance.

C.E./N.E: cancer/normal tissue from ever smokers; C.N/N.N: cancer/normal tissue from never smokers;

**Table.S33 Significantly different microbes between younger and older patients. (age < 50; age ≥ 50)**

| **Taxa** | **Mean relative abundance** | | | |  | **P.value^a^** | **FDR** |
| --- | --- | --- | --- | --- | --- | --- | --- |
| **Genus** | Mean (patient age < 50) | SD | Mean (patient age ≥ 50) | SD |  |  |  |
| Acetobacter | 0.080 | 0.11 | 0.025 | 0.053 |  | 0.037 | 0.30 |
| Acinetobacter | 8.9 | 1.2 | 7.4 | 1.2 |  | 0.0049 | 0.20 |
| Actinomyces | 0.096 | 0.13 | 0.32 | 0.36 |  | 0.031 | 0.30 |
| Asticcacaulis | 0.27 | 0.20 | 0.13 | 0.12 |  | 0.041 | 0.30 |
| Clostridium | 1.2 | 0.41 | 0.85 | 0.32 |  | 0.033 | 0.30 |
| Corynebacterium | 1.2 | 0.41 | 1.8 | 0.80 |  | 0.033 | 0.30 |
| Deinococcus | 0.20 | 0.17 | 0.38 | 0.25 |  | 0.033 | 0.30 |
| Fusobacterium | 0.031 | 0.058 | 0.39 | 1.0 |  | 0.0062 | 0.20 |
| Halorhabdus | 0.071 | 0.090 | 0.011 | 0.038 |  | 0.021 | 0.30 |
| Lactococcus | 0.16 | 0.089 | 0.076 | 0.091 |  | 0.024 | 0.30 |
| Mogibacterium | 0.43 | 0.21 | 0.31 | 0.13 |  | 0.037 | 0.30 |
| Mycoplasma | 0.024 | 0.071 | 1.2 | 2.5 |  | 0.016 | 0.30 |
| Oscillospira | 1.1 | 0.29 | 0.80 | 0.20 |  | 0.0042 | 0.20 |

a: analyzed using combined 16S data; wilcox.test, P<0.05 set for genus.

**Table.S34 Significantly different microbes found in patients’ anatomy site. (****Left lung upper/lower; Right lung upper/lower)**

| **Taxa** | **Mean relative abundance** | | | | | | | | **P.value^a^** | **FDR** |
| --- | --- | --- | --- | --- | --- | --- | --- | --- | --- | --- |
| **Genus** | Mean (L_L) | SD (L_L) | Mean (L_U) | SD (L_U) | Mean (R_L) | SD (R_L) | Mean (R_U) | SD (R_U) |  |  |
| Aggregatibacter | 0.0094 | (0.025) | 0.075 | (0.010) | 0.27 | (0.25) | 0.034 | (0.095) | 0.012 | 0.31 |
| Akkermansia | 0.049 | (0.057) | 0.24 | (0.15) | 0.17 | (0.091) | 0.12 | (0.11) | 0.030 | 0.43 |
| Alicyclobacillus | 0.091 | (0.069) | 0.26 | (0.12) | 0.31 | (0.18) | 0.12 | (0.13) | 0.013 | 0.31 |
| Aquicella | 0 | (0) | 0.10 | (0.10) | 0 | (0) | 0.024 | (0.068) | 0.0030 | 0.29 |
| Blastomonas | 0.011 | (0.021) | 0.054 | (0.063) | 0.20 | (0.14) | 0.15 | (0.18) | 0.031 | 0.43 |
| Capnocytophaga | 0.069 | (0.092) | 0.0086 | (0.023) | 0.48 | (0.85) | 0.043 | (0.11) | 0.021 | 0.40 |
| Cellvibrio | 0.25 | (0.15) | 0.097 | (0.099) | 0.30 | (0.19) | 0.25 | (0.11) | 0.047 | 0.54 |
| Novosphingobium | 0.0021 | (0.0055) | 0.032 | (0.061) | 0.0064 | (0.017) | 0.16 | (0.16) | 0.012 | 0.31 |

a: analyzed using combined 16S data; kruskal.test, P<0.05 set for genus.

L_L: left lung lower lobe; L_U: left lung upper lobe; R_L: right lung lower lobe; R_U: right lung upper lobe.

**Table.S35 Significantly different microbes between patients’ blood type.**

| **Taxa** | **Mean relative abundance** | | | | | | | | **P.value^a^** | **FDR** |
| --- | --- | --- | --- | --- | --- | --- | --- | --- | --- | --- |
| **Genus** | Mean (A) | SD (A) | Mean (AB) | SD (AB) | Mean (B) | SD (B) | Mean (O) | SD (O) |  |  |
| Brachybacterium | 0.16 | (0.15) | 0.53 | (0.14) | 0.083 | (0.075) | 0.12 | (0.098) | 0.034 | 0.93 |
| Dyadobacter | 0.0029 | (0.0081) | 0.11 | (0.040) | 0.048 | (0.12) | 0.017 | (0.041) | 0.020 | 0.93 |

a: analyzed using combined 16S data; kruskal.test, P<0.05 set for genus.

**Table.S36 Significantly different microbes between AD and SCC.**

| **Taxa** | **Mean relative abundance** | | | |  | **P.value^a^** | **FDR** |
| --- | --- | --- | --- | --- | --- | --- | --- |
| **Genus** | Mean (AD) | SD (AD) | Mean (SCC) | SD (SCC) |  |  |  |
| Aerococcus | 0.79 | 0.27 | 0.47 | 0.28 |  | 0.0039 | 0.19 |
| Brevibacterium | 0.32 | 0.18 | 0.081 | 0.12 |  | 0.0034 | 0.19 |
| Capnocytophaga | 0.21 | 0.52 | 0 | 0 |  | 0.0074 | 0.24 |
| Finegoldia | 0.067 | 0.13 | 0 | 0 |  | 0.044 | 0.64 |
| Oligella | 0.17 | 0.11 | 0.12 | 0.24 |  | 0.034 | 0.64 |

a: analyzed using combined 16S data; wilcox.test, P<0.05 set for genus.

AD: Adenocarcinoma; SCC: squamous cell carcinoma.

**Table.S37 Significantly different microbes between T1_2 and T3_4. (T stage of tumor TNM stage)**

| **Taxa** | **Mean relative abundance** | | | |  | **P.value^a^** | **FDR** |
| --- | --- | --- | --- | --- | --- | --- | --- |
| **Genus** | Mean (T1_2) | SD (T1_2) | Mean (T3_4) | SD (T3_4) |  |  |  |
| Alicyclobacillus | 0.15 | 0.12 | 0.26 | 0.17 |  | 0.049 | 0.75 |
| Capnocytophaga | 0.018 | 0.041 | 0.28 | 0.62 |  | 0.028 | 0.75 |
| Haemophilus | 0.051 | 0.083 | 0.23 | 0.32 |  | 0.013 | 0.65 |
| Lactobacillus | 1.1 | 0.29 | 0.80 | 0.37 |  | 0.0030 | 0.29 |
| Massilia | 0.53 | 0.55 | 0.28 | 0.16 |  | 0.044 | 0.75 |
| Neisseria | 0.039 | 0.061 | 0.23 | 0.33 |  | 0.037 | 0.75 |

a: analyzed using combined 16S data; wilcox.test, P<0.05 set for genus.

**Table.S38 Significantly different microbes between N0 and N1_2. (N stage of tumor TNM stage)**

| **Taxa** | **Mean relative abundance** | | | |  | **P.value^a^** | **FDR** |
| --- | --- | --- | --- | --- | --- | --- | --- |
| **Genus** | Mean (N0) | SD (N0) | Mean (N1_2) | SD (N1_2) |  |  |  |
| Bacteroides | 0.22 | 0.14 | 0.31 | 0.80 |  | 0.032 | 0.34 |
| Blastomonas | 0.14 | 0.15 | 0.058 | 0.11 |  | 0.030 | 0.34 |
| Bradyrhizobium | 0.21 | 0.14 | 0.088 | 0.077 |  | 0.012 | 0.34 |
| Clostridium | 0.80 | 0.31 | 1.1 | 0.38 |  | 0.023 | 0.34 |
| Corynebacterium | 1.5 | 0.87 | 1.8 | 0.51 |  | 0.023 | 0.34 |
| Eikenella | 0.0096 | 0.024 | 0.11 | 0.16 |  | 0.038 | 0.36 |
| Kaistobacter | 0.13 | 0.20 | 0.020 | 0.033 |  | 0.017 | 0.34 |
| Lachnoanaerobaculum | 0 | 0 | 0.039 | 0.078 |  | 0.0092 | 0.34 |
| Leptotrichia | 0.084 | 0.12 | 0.46 | 0.52 |  | 0.030 | 0.34 |
| Neisseria | 0.035 | 0.063 | 0.24 | 0.33 |  | 0.0061 | 0.34 |

a: analyzed using combined 16S data; wilcox.test, P<0.05 set for genus.

**Table.S39 Significantly different microbes between Stage I_II and Stage III_IV. (Stage of tumor TNM stage)**

| **Taxa** | **Mean relative abundance** | | | |  | **P.value^a^** | **FDR** |
| --- | --- | --- | --- | --- | --- | --- | --- |
| **Genus** | Mean (Stage I_II) | SD (Stage I_II) | Mean (Stage III_IV) | SD (Stage III_IV) |  |  |  |
| Alicyclobacillus | 0.13 | 0.11 | 0.26 | 0.16 |  | 0.018 | 0.50 |
| Blautia | 0.11 | 0.099 | 0.032 | 0.060 |  | 0.0098 | 0.50 |
| Cloacibacterium | 0.17 | 0.24 | 0.037 | 0.070 |  | 0.049 | 0.50 |
| Haemophilus | 0.046 | 0.077 | 0.20 | 0.30 |  | 0.035 | 0.50 |
| Lachnoanaerobaculum | 0 | 0 | 0.032 | 0.072 |  | 0.033 | 0.50 |
| Lactobacillus | 1.1 | 0.26 | 0.86 | 0.40 |  | 0.015 | 0.50 |
| Leptotrichia | 0.073 | 0.10 | 0.40 | 0.49 |  | 0.044 | 0.50 |
| Neisseria | 0.037 | 0.065 | 0.20 | 0.31 |  | 0.037 | 0.50 |

a: analyzed using combined 16S data; wilcox.test, P<0.05 set for genus.
