# Supplementary material for: A Multi-Omics Study of Familial Lung Cancer: Microbiome and Host Gene Expression Patterns
Source: Front Immunol. 2022 Apr 11;13:827953. doi: 10.3389/fimmu.2022.827953 (PMC9037597; doi:10.3389/fimmu.2022.827953)
Supplement: Supplementary Tables 40–42 — for significantly different top genes between groups; [file DataSheet_4.docx]

**Table.S40-S42 for significantly different top genes between groups.**

**Table.S40 Significantly different top 10 Genes between FLC and Sporadic group**

| **Gene** | **Relative expression** | | | **P.value^a^** | **Kegg Note** |
| --- | --- | --- | --- | --- | --- |
|  | FLC | Sporadic | Up-Down (F/S) |  |  |
| RND1 | 16233 | 20973 | Down | < 2.00E-259 | K07531//Rho family GTPase 1 |
| CHI3L1 | 22942 | 5858 | Up | < 2.00E-259 | K17523//chitinase-3-like protein 1/2 |
| CEACAM5 | 5547.07 | 8178.96 | Down | < 2.00E-259 | K06499//carcinoembryonic antigen-related cell adhesion molecule |
| CCL20 | 855 | 3190 | Down | < 2.00E-259 | K14625//C-C motif chemokine 20 |
| DEFA1B | 5857.66 | 665.57 | Up | < 2.00E-259 | K05230//defensin, alpha |
| NTRK2 | 16477 | 4603 | Up | < 2.00E-259 | K04360//neurotrophic tyrosine kinase receptor type 2 [EC:2.7.10.1] |
| ARC | 11098 | 2763 | Up | < 2.00E-259 | K15867//activity-regulated cytoskeleton-associated protein |
| CORO7-PAM16 | 204.2 | 1474.23 | Down | < 2.00E-259 | K18619//coronin-7 |
| MMP9 | 10416 | 2803 | Up | < 2.00E-259 | K01403//matrix metalloproteinase-9 (gelatinase B) [EC:3.4.24.35] |
| KRT16 | 4250.07 | 5715.31 | Down | < 2.00E-259 | K07604//type I keratin, acidic |

a: Top genes were listed in the table, all selected with the P < 2.00E-259, the exact number were not shown.

FLC: Familial lung cancer; Sporadic: Sporadic lung cancer

**Table.S41 Significantly different top 10 Genes between High-IAP and Low-IAP group**

| **Gene** | **Relative expression** | | | **P.value^a^** | **Kegg Note** |
| --- | --- | --- | --- | --- | --- |
|  | High-IAP | Low-IAP | Up-Down (H/L) |  |  |
| IGLL5 | 250384.88 | 88901.94 | Up | < 8.54E-308 | K06554//immunoglobulin lambda-like polypeptide 1 |
| CSF3 | 40652.97 | 6908 | Up | < 8.54E-308 | K05423//granulocyte colony-stimulating factor |
| HP | 13533.95 | 32776.55 | Down | < 8.54E-308 | K16142//haptoglobin |
| ITPKC | 53762 | 16643 | Up | < 8.54E-308 | K00911//1D-myo-inositol-triphosphate 3-kinase [EC:2.7.1.127] |
| ITLN1 | 14718.38 | 31501.53 | Down | < 8.54E-308 | K17527//intelectin |
| RND1 | 27316 | 9890 | Up | < 8.54E-308 | K07531//Rho family GTPase 1 |
| SERPINA3 | 18428 | 6428 | Up | < 8.54E-308 | K04525//serpin peptidase inhibitor, clade A |
| IL1RL1 | 19546.69 | 7162.7 | Up | < 8.54E-308 | K05171//interleukin 1 receptor-like 1 |
| DEFA1B | 1369.27 | 5153.96 | Down | < 8.54E-308 | K05230//defensin, alpha |
| SGPP2 | 18294.98 | 6956.52 | Up | < 8.54E-308 | K04717//sphingosine-1-phosphate phosphotase 2 [EC:3.1.3.-] |

a: Top genes were listed in the table, selected with the P < 8.54E-308, the exact number were not shown.

H: High-IAP; L: Low-IAP; IAP: indoor air pollution

**Table.S42 Significantly different top 10 Genes between Female and Male group**

| **Gene** | **Relative expression** | | | **P.value^a^** | **Kegg Note** |
| --- | --- | --- | --- | --- | --- |
|  | Female | Male | Up-Down (F/M) |  |  |
| DDX3Y | 49.53 | 33006.14 | Down | < 1.15E-302 | K17642//ATP-dependent RNA helicase DDX3Y [EC:3.6.4.13] |
| CCL18 | 17360 | 82041.35 | Down | < 1.15E-302 | K21094//C-C motif chemokine 18 |
| HP | 5332.5 | 40978 | Down | < 1.15E-302 | K16142//haptoglobin |
| CSF3 | 27358 | 20202.97 | Up | < 1.15E-302 | K05423//granulocyte colony-stimulating factor |
| USP9Y | 10.03 | 8127.53 | Down | < 1.15E-302 | K11840//ubiquitin carboxyl-terminal hydrolase 9/24 [EC:3.4.19.12] |
| ITLN1 | 10401.12 | 35818.79 | Down | < 1.15E-302 | K17527//intelectin |
| MMP9 | 8834 | 4385 | Up | < 1.15E-302 | K01403//matrix metalloproteinase-9 (gelatinase B) [EC:3.4.24.35] |
| UTY | 686.73 | 8073.03 | Down | < 1.15E-302 | K11447//histone demethylase [EC:1.14.11.-] |
| CCL14 | 14378 | 10552 | Up | < 1.15E-302 | K21092//C-C motif chemokine 14 |
| CYP1B1 | 5206.57 | 18525.81 | Down | < 1.15E-302 | K07410//cytochrome P450 family 1 subfamily B polypeptide 1 [EC:1.14.14.1] |

a: Top genes were listed in the table, selected with the P < 1.15E-302, the exact number were not shown.

F: female; M: male;
