# Supplementary figures and images for: A Multi-Omics Study of Familial Lung Cancer: Microbiome and Host Gene Expression Patterns
Source: Front Immunol. 2022 Apr 11;13:827953. doi: 10.3389/fimmu.2022.827953 (PMC9037597; doi:10.3389/fimmu.2022.827953)

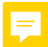

Supplement: Supplementary file 6 [file Image_1.pdf]

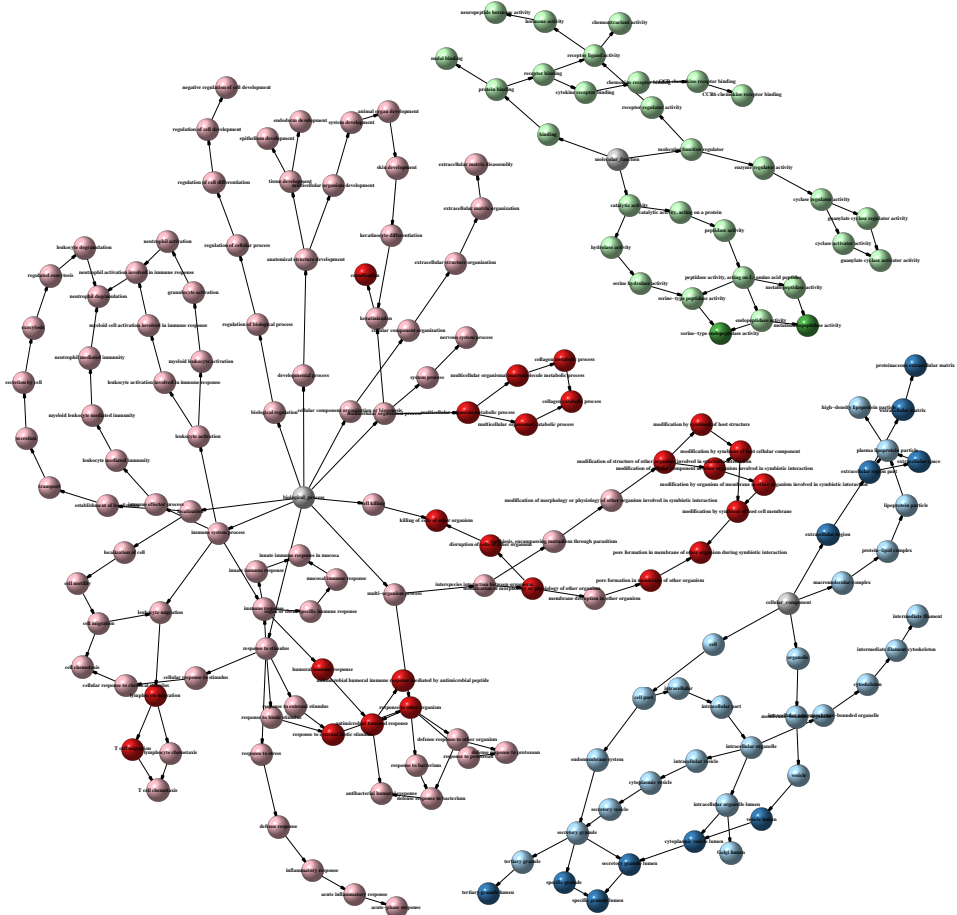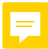

Supplement: Supplementary file 7 [file Image_2.pdf]

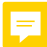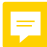

Supplement: Supplementary file 8 [file Image_3.pdf]
